# Nonlinear effects of post-denudation timing on day 3 embryo outcomes in ICSI and evidence for a translatable optimization window

**DOI:** 10.1186/s12967-026-08586-0

**Published:** 2026-07-11

**Authors:** Yongfang Xu, Yunxiu Li, Yonggang Li, Lifeng Xiang, Haishan Zheng, Mengying Gao, Lian Deng, Mingying Li, Peng Zeng, Xiaorong Wu, Manqin Zhang, Lei Ding, Ze Wu, Xi Luo

**Affiliations:** 1 Department of Reproductive Medicine, NHC Key Laboratory of Healthy Birth and Birth Defect Prevention in Western China, The First People’s Hospital of Yunnan Province, Kunming, 650032 China; 2 Reproductive Medical Center of Yunnan Province, The Affiliated Hospital of Kunming University of Science and Technology, Kunming, 650032 China

**Keywords:** Intracytoplasmic sperm injection, Oocyte denudation, Embryo development, Restricted cubic spline analysis, Assisted reproductive technology

## Abstract

**Background:**

Intracytoplasmic sperm injection (ICSI) is performed in over half of assisted reproductive technology cycles globally; however, the optimal interval between oocyte denudation and sperm injection remains undefined in clinical guidelines. This knowledge gap hinders the standardization of laboratory workflows and may compromise embryo developmental potential due to suboptimal timing practices. It necessitates a comprehensive investigation to formulate evidence-based recommendations.

**Methods:**

This retrospective cohort study examined 1,152 consecutive fresh ICSI cycles performed at an academic reproductive center between January 2024 and April 2025 to establish quantitative timing guidance. The denudation-to-ICSI interval (DTI) was defined as the primary exposure, and the day-3 embryo utilization rate, calculated as the number of usable embryos divided by the number of retrieved oocytes, was designated as the primary outcome. Nonlinear dose-response relationships were evaluated using generalized additive models and restricted cubic spline analyses, with multivariable adjustment for demographic characteristics, ovarian reserve parameters, and treatment-related factors. Effect heterogeneity was examined across 19 predefined patient subgroups, and robustness of the findings was validated through bootstrap resampling and cross-validation.

**Results:**

A significant inverted U-shaped relationship was identified between DTI and day 3 embryo utilization rate. In the final parsimonious model adjusted for basal FSH and E2 per MII (*p* = 0.017), predicted utilization rates spanned 12.2 percentage points across the observed DTI range, peaking at 2.75 h post-denudation and defining an optimal window of 1.75–3.75 h. This association was robust to progressive covariate adjustment (24% coefficient attenuation from univariate to fully adjusted model, *p* = 0.036) and consistent across all 19 patient subgroups (no significant interactions; I^2^ < 35%). The timing effect was specific to day 3 embryo development without influencing fertilization or cleavage rates. Approximately 30% of current cycles fell outside the optimal window, corresponding to 4.6 percentage points lower utilization.

**Conclusions:**

This study identified an optimal window of 1.75–3.75 h for ICSI after oocyte denudation, with consistent associations observed across diverse patient subgroups within the studied population. The identified window represents a workflow-level optimization achievable through procedural standardization alone, with the potential to increase embryo availability for a substantial proportion of cycles currently outside this timeframe. Future trials are warranted to confirm causality and measure improvements in clinical pregnancy outcomes.

**Clinical trial number:**

Not applicable.

**Supplementary Information:**

The online version contains supplementary material available at 10.1186/s12967-026-08586-0.

## Background

Intracytoplasmic sperm injection (ICSI) has become an indispensable component of assisted reproductive technology (ART), accounting for approximately 50–70% of in vitro fertilization cycles worldwide [1, 2]. As the primary treatment for severe male factor infertility and fertilization failure, the technique has undergone three decades of refinement. Essential elements of laboratory procedures, particularly the optimal temporal sequence from oocyte denudation to sperm injection, remain insufficiently defined [3]. Without evidence-based timing guidelines, individual laboratories adhere to institutional convention instead of optimal protocols, potentially compromising embryo outcomes for substantial patient populations.

The biological rationale for timing sensitivity is widely recognized. Oocyte denudation, a prerequisite for ICSI, involves enzymatic removal of cumulus cells using hyaluronidase [4]. This removal disrupts metabolic and paracrine signaling critical for oocyte competence [5, 6]. Cumulus cells provide pyruvate, lactate, amino acids, and antioxidant protection through glutathione transfer [7, 8]. Following denudation, the lack of gap junctional communication impairs glucose metabolism and ATP synthesis [9], whereas removal of antioxidant defense renders oocytes vulnerable to reactive oxygen species, potentially causing mitochondrial dysfunction and DNA damage [10]. Cytoplasmic maturation activities may persist during this period, as demonstrated in mammalian oocyte models [11], yet extended durations accelerate oocyte aging through oxidative damage [12]. The interplay between potential maturation advantage and aging risk thus suggests the existence of a nonlinear dose-response relationship with an identifiable optimal window.

The existing literature exhibits conflicting evidence that has hindered translation into clinical practice. A systematic review revealed inconsistent and contradictory findings regarding time intervals between oocyte retrieval, denudation, and injection [13], but a large cohort study indicated no significant timing effects on ICSI outcomes [14]. The current ESHRE guidelines address oocyte insemination procedures [15] but offer limited empirical justification for specific time thresholds, leaving practitioners without actionable recommendations. This translational gap between laboratory observations and clinical standardization remains unresolved.

These contradictions likely stem from significant methodological limitations, including categorical time groupings that obscure nonlinear relationships, heterogeneous outcome measures, and insufficient confounder adjustment. A systematic assessment of dose-response relationships and effect heterogeneity across patient subgroups categorized by age, ovarian reserve, and response patterns is lacking. The relationship between the denudation-to-ICSI interval (DTI) and other critical time intervals, including trigger-to-retrieval and retrieval-to-denudation, also remains unaddressed, leaving potential confounding unresolved.

We therefore conducted a retrospective cohort study encompassing 1,152 consecutive ICSI cycles. The primary aim was to characterize the DTI-embryo utilization relationship using advanced nonlinear modeling approaches, notably generalized additive models and restricted cubic splines (RCS). Secondary objectives were to assess the effects on embryo quality, fertilization, and cleavage rates; to examine effect heterogeneity among patient subgroups; and to define a data-driven optimal operating window suitable for clinical consideration. We hypothesized that a nonlinear, inverted U-shaped relationship exists, with a discernible optimal time point that balances maturation benefits against aging risks. Such a relationship, if robust across diverse populations, could be incorporated into standardized laboratory protocols.

## Materials and methods

### Study design and setting

This retrospective cohort study examined consecutive ICSI cycles performed between January 2024 and April 2025 at the Department of Reproductive Medicine, Affiliated Hospital of Kunming University of Science and Technology, a tertiary academic reproductive center in Kunming, Yunnan Province, southwestern China. The center serves a demographically diverse population, including Han Chinese (61.7%) and ethnic minority groups (38.3%), reflecting the multiethnic composition of the region. The study protocol was approved by the institutional review board with approval no. KHLL2025-KY373. Data were extracted from the electronic medical record system with complete anonymization. All procedures followed ESHRE guidelines for good practice in in vitro fertilization (IVF) laboratories.

### Study population

The study included all fresh ICSI cycles with planned (non-rescue) procedures during the study period. The inclusion criteria comprised women aged 20–50 years receiving treatment with autologous fresh oocytes, completion of the entire laboratory process from oocyte retrieval through denudation to ICSI injection, at least one mature oocyte successfully injected, and continuation of embryo culture to day 3 with morphological assessment.

The exclusion criteria included donor sperm usage, in vitro maturation protocols, rescue ICSI after failed conventional IVF, and cycles with missing critical time points that hindered the calculation of the DTI or resulted in logical discrepancies in time sequence. Cycles leading to fertilization failure or early developmental arrest were retained, as these outcomes may reflect biologically relevant effects of timing. Patients with controlled endocrine disorders, uterine abnormalities, tubal disease, endometriosis, diminished ovarian reserve, or male factor infertility were included to enhance the real-world applicability of the findings.

### Clinical and laboratory procedures

Controlled ovarian stimulation utilized standard protocols, including the GnRH agonist long protocol, the GnRH antagonist protocol, mild stimulation, and natural cycle, with protocol selection based on patient characteristics. Follicular development was assessed through transvaginal ultrasound and serum estradiol levels. Final oocyte maturation was induced with recombinant hCG when a minimum of two follicles reached 18 mm in diameter, with triggering time recorded electronically.

Oocyte pickup (OPU) was performed 34 to 38 h post-trigger under ultrasound guidance, with the exact retrieval time recorded. All procedural timestamps were captured through a mandatory patient identity verification step embedded in the laboratory workflow, with each entry constituting an uneditable original record. Following retrieval, cumulus-oocyte complexes were cultured in standard IVF medium at 37 °C in an atmosphere of 6% CO_2_ until denudation. Denudation was performed using hyaluronidase (80 IU/mL, 30 s), followed by gentle mechanical pipetting. The completion time of denudation was recorded once all cumulus cells had been removed and oocyte maturity could be assessed. Mature MII oocytes, identified by the presence of the first polar body and the absence of the germinal vesicle, were selected for injection. ICSI was performed by experienced embryologists (≥5 years of practice) operating under a daily rotation system, with case allocation independent of patient complexity or prognosis. Operator performance was monitored through routine quality control assessments to maintain procedural consistency across the team. The injection time was recorded immediately upon completion of sperm deposition.

Fertilization was evaluated 16 to 18 h post-ICSI on day 1, with normal fertilization characterized by the presence of two pronuclei and two polar bodies. Embryo cleavage was evaluated on day 2 at 44 ± 1 h post-ICSI. Day 3 embryo quality assessment was performed at 68 ± 1 h post-ICSI using standardized morphological criteria. Embryos were graded A to D based on blastomere number, symmetry, and fragmentation percentage. Grade A embryos contained 8 cells with complete symmetry and <5% fragmentation. Grade B embryos contained 7–9 cells with slight asymmetry and 5–10% fragmentation. Grade C embryos contained 6–10 cells with mild-moderate asymmetry and >10%, ≤20% fragmentation. Grade D embryos contained abnormal cell numbers, severe asymmetry, >20% fragmentation, or developmental arrest.

### Study variables and outcomes

The primary exposure variable was the DTI, calculated as the time elapsed between denudation completion and ICSI injection, measured in hours with minute-level accuracy. Secondary time intervals included trigger-to-retrieval and retrieval-to-denudation intervals, examined as potential confounders or impact modifiers.

The primary outcome was the day 3 embryo utilization rate, defined as the number of usable embryos (grades A, B, and C) divided by the total number of retrieved oocytes, thereby reflecting fertilization efficiency and early embryonic development potential. Secondary outcomes included good quality embryo rate (grades A and B divided by number of retrieved oocytes), the normal fertilization rate (two-pronuclear zygotes divided by the number of injected oocytes), and cleavage rate (cleaved embryos on day 2 divided by injected oocytes).

Baseline patient characteristics were collected across five domains. Demographic variables included patient physical attributes and ethnic background. Reproductive history captured previous pregnancy outcomes and infertility patterns. Ovarian reserve markers evaluated ovarian function and follicular development potential. Treatment parameters recorded stimulation protocols and hormonal responses. Laboratory outcomes encompassed oocyte yield and early embryonic development metrics.

### Statistical software and general approach

All statistical analyses were performed using R software (version 4.4.1). RCS analyses were conducted with the rms package (version 8.0.0) [16], generalized additive models were fitted using mgcv (version 1.9.3) [17], and elastic net regularization was implemented with glmnet (version 4.1.10) [18]. Bootstrap validation was performed using boot (version 1.3.32) [19], a cross-validation procedure was conducted using caret (version 7.0.1) [20], and statistical power analysis was conducted using pwr (version 1.3.0) [21]. Model diagnostics were assessed using the car (version 3.1.3) and lmtest (version 0.9.40) [22, 23]. Data cleaning and processing were conducted using the tidyverse ecosystem [24].

Statistical significance was set at two-sided α = 0.05 for confirmatory analyses and α = 0.10 for exploratory interaction testing. Effect estimates are presented with 95% confidence intervals (CI). Model performance was evaluated using Akaike information criterion (AIC) and Bayesian information criterion (BIC), with lower values indicating improved model fit. Predictive performance was assessed using root mean square error (RMSE) and mean absolute error (MAE). Heterogeneity was quantified using I^2^ statistics, with values < 25%, 25–50%, and >50% indicating low, moderate, and high heterogeneity, respectively. Post-hoc power analysis was conducted using Cohen’s f^2^ effect size to assess sample size adequacy for detecting observed effects, with statistical power exceeding 70% considered sufficient for reliable inference.

### Statistical modeling

Data preprocessing included comprehensive quality control procedures, encompassing missing value assessment, outlier detection using the interquartile range method (coefficient of 3), and logical consistency verification. Descriptive statistics are presented as mean ± SD for normally distributed variables or median (interquartile range [IQR]) for skewed distributions, with categorical variables expressed as frequencies and percentages.

The association between DTI and embryo utilization rate was initially explored using generalized additive models (GAM) to identify potential nonlinear patterns without parametric assumptions. Variables exhibiting evidence of nonlinearity, defined as effective degrees of freedom (EDF) >1.5 with statistical significance, were subsequently modeled using RCS. The optimal number of spline knots was determined by minimizing BIC, with candidate models evaluated using 3- to 7-knot configurations. Knot positions were determined using the percentile-based placement strategy recommended by Harrell [16], with the 3-knot model placing knots at the 10th, 50th, and 90th percentiles of the exposure distribution. Given the inherent correlations among sequential time intervals, independence was assessed using VIF analysis (threshold < 5), and potential interaction effects were evaluated using likelihood ratio tests. The effects of secondary time intervals on other laboratory outcomes were examined in separate GAM analyses.

Covariate selection commenced with multicollinearity diagnostics to exclude highly correlated predictors, followed by univariate screening in which variables with *p* < 0.20 or established clinical relevance were retained. A progressive model-building strategy was employed, in which Model 1 included DTI alone, Model 2 added demographic factors, Model 3 incorporated ovarian reserve markers, and Model 4 included treatment variables. Time effect stability was tracked across models to assess potential confounding.

Final model optimization employed a dual-criteria selection strategy combining BIC-based backward stepwise elimination and elastic net regularization (α = 0.5). Variables identified by both methods with consistency > 70% were retained. Model comparison utilized AIC, BIC, and adjusted R^2^. Comprehensive model diagnostics were conducted, including residual analysis, leverage assessment, and influence diagnostics using Cook’s distance.

### Model validation and clinical application

Heterogeneity of the DTI effect was assessed using tiered interaction testing. Primary interactions within the final model were tested first, followed by interactions with age groups and anti-Müllerian hormone (AMH) categories, and subsequently with other time intervals. Multiple comparisons were adjusted using the false discovery rate method. Subgroup analyses were performed for all patient strata with ≥30 cycles per subgroup; stratum-specific estimates were reported with 95% CI. Statistical heterogeneity was quantified using Cochran’s Q test and I^2^ statistics, with between-study variance (τ^2^) estimated using random-effects models.

Comprehensive sensitivity analyses were conducted to evaluate model robustness. Bootstrap resampling with 2,000 iterations was performed to assess coefficient stability and 95% CI consistency. Influential observations, identified using Cook’s distance > 4/n, were systematically excluded. Extreme value handling included winsorization at the 1st and 5th percentiles. Where appropriate, heteroscedasticity-consistent robust standard errors (HC3) were applied. Predictive stability was further evaluated using leave-one-out cross-validation and 10-fold cross-validation (5 repetitions), with R^2^, RMSE, and MAE used as performance metrics.

Optimal ICSI timing was determined using dual strategies. In strategy A, fixed median covariate values were applied to predict utilization rates across the observed DTI range. Strategy B used bootstrap sampling from the 40th to 60th percentile covariate ranges (500 iterations). Convergence, defined as differences in the estimated optimum, indicated robust optimal timing. Clinical operating windows were defined using performance thresholds relative to peak predicted utilization, considering population coverage, window width, and expected outcomes. Time segment analysis further divided the continuous range into 30-min intervals, comparing mean utilization rates using t-tests with Bonferroni correction to confirm continuous model predictions.

## Results

### Study population and baseline characteristics

**Fig. 1:**
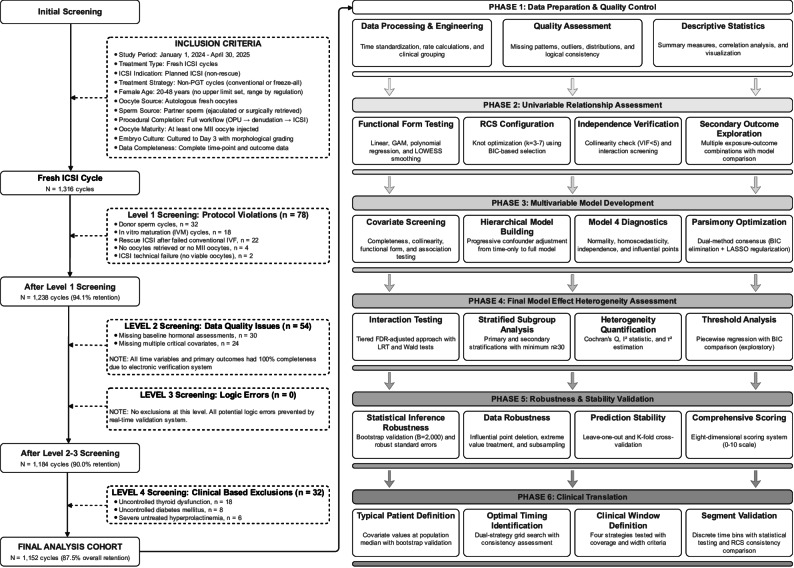
Comprehensive study design flowchart illustrating patient selection process and six-phase analytical workflow. Study flowchart with dual components. The left panel illustrates sequential patient screening through four exclusion levels, from 1,316 initial fresh ICSI cycles to the final analytical cohort of 1,152 cycles (87.5% retention). The right panel presents the complete methodological framework across six analytical phases: data preparation, univariable assessment, multivariable model development, heterogeneity evaluation, robustness validation, and clinical translation, with key methods specified for each phase

We analyzed 1,152 consecutive, unselected ICSI cycles performed between January 2024 and April 2025 (Fig. 1), reflecting routine clinical practice without patient selection or outcome restrictions. Table 1 presents patient demographics, clinical characteristics, treatment parameters, and laboratory outcomes.

**Table 1:** Baseline demographic, clinical, and laboratory characteristics of the study population

Category	Variable	N	Mean ± SD	Median [IQR]	Range
**Demographics**	Female age, years	1152	32.66 ± 5.47	32 [29, 36]	[20, 50]
	Male age, years	1152	34.22 ± 5.74	34 [30, 37]	[22, 54]
	BMI, kg/m^2^	1152	22.56 ± 3.07	22.40 [20.40, 24.60]	[15.60, 36.50]
	Age at menarche, years	1152	13.09 ± 0.57	13 [13, 13]	[11, 17]
	Female ethnicity	1152	—	—	—
	*Han*	711 (61.7%)	—	—	—
	*Minority*	441 (38.3%)	—	—	—
	Male ethnicity	1152	—	—	—
	*Han*	760 (66%)	—	—	—
	*Minority*	392 (34%)	—	—	—
**Reproductive History**	Infertility duration, years	1152	4.02 ± 3.24	3 [2, 5]	[1, 25]
	Infertility type	1152	—	—	—
	*Primary*	682 (59.2%)	—	—	—
	*Secondary*	470 (40.8%)	—	—	—
	Infertility diagnosis	1152	—	—	—
	*Male factor only*	347 (30.1%)	—	—	—
	*Tubal factor*	279 (24.2%)	—	—	—
	*Ovulatory disorder*	122 (10.6%)	—	—	—
	*Uterine factor*	141 (12.2%)	—	—	—
	*Unexplained infertility*	26 (2.3%)	—	—	—
	*Other factors*	135 (11.7%)	—	—	—
	*Combined factors*	102 (8.9%)	—	—	—
	Gravidity	1152	0.78 ± 1.21	—	[0, 8]
	Parity	1152	0.28 ± 0.62	—	[0, 4]
	Abortions, n	1152	0.28 ± 0.72	—	[0, 6]
	Miscarriages, n	1152	0.14 ± 0.46	—	[0, 5]
	Ectopic pregnancies, n	1152	0.08 ± 0.34	—	[0, 3]
	ART cycle, n	1152	1.76 ± 1.29	1 [1, 2]	[1, 12]
**Ovarian Reserve**	AMH, ng/mL	1152	3.66 ± 2.94	2.90 [1.60, 4.80]	[0.10, 18.00]
	AFC	1152	13.99 ± 8.05	13 [8, 18]	[0, 47]
	Basal FSH, IU/L	1152	7.01 ± 3.73	6.70 [4.80, 8.40]	[0.70, 23.40]
	Basal LH, IU/L	1152	4.76 ± 3.12	4.40 [2.30, 6.20]	[0.60, 18.30]
	Basal E2, pg/mL	1152	116.38 ± 89.97	105 [37, 154]	[18, 492]
	TSH, mIU/L	1152	2.35 ± 0.94	2.26 [1.60, 3.01]	[0.52, 4.99]
**Stimulation & Treatment**	Stimulation protocol	1152	—	—	—
	*Agonist*	428 (37.2%)	—	—	—
	*Antagonist*	340 (29.5%)	—	—	—
	*Progestin-primed*	357 (31%)	—	—	—
	*Mild*	27 (2.3%)	—	—	—
	Stimulation duration, days	1152	9.84 ± 1.98	10 [9, 11]	[2, 22]
	Total Gn dose, IU	1152	1722.36 ± 649.49	1650 [1225, 2100]	[150, 4650]
	E2 at trigger, pg/mL	1152	8944.57 ± 6189.10	7788 [4727, 10,950]	[165, 47,494]
	Sperm source	1152	—	—	—
	*Ejaculated*	838 (72.7%)	—	—	—
	*Surgical*	314 (27.3%)	—	—	—
**Laboratory Outcomes**	Oocytes retrieved, n	1152	11.50 ± 7.37	10 [6, 16]	[1, 43]
	E2 per oocyte, pg/mL	1152	901.77 ± 503.97	786.49 [593.42, 1068.36]	[41.25, 7019.00]
	MII oocytes, n	1152	9.04 ± 5.91	8 [4, 12]	[1, 36]
	E2 per MII, pg/mL	1152	1177.45 ± 812.82	995.82 [751.48, 1373.14]	[41.25, 11,914.00]
	Immature oocytes, n	1152	2.46 ± 2.76	2 [0, 3]	[0, 33]
	MII rate	1152	0.81 ± 0.17	0.83 [0.71, 1.00]	[0.10, 1.00]
	Oocytes injected, n	1152	9.04 ± 5.91	8 [4, 12]	[1, 36]
	2 PN, n	1152	6.89 ± 4.99	6 [3, 10]	[0, 30]
	Fertilization rate (2 PN)	1152	0.75 ± 0.24	0.80 [0.67, 0.93]	[0.00, 1.00]
	Cleaved embryos (D2), n	1152	7.22 ± 5.14	6 [3, 10]	[0, 29]
	Cleavage rate	1152	0.79 ± 0.22	0.85 [0.70, 1.00]	[0.00, 1.00]
	D3 usable embryos (A+B+C), n	1152	5.24 ± 4.18	4 [2, 7]	[0, 23]
	D3 good quality embryos (A+B), n	1152	1.85 ± 1.98	1 [0, 3]	[0, 13]
	D3 embryo utilization rate	1152	0.48 ± 0.26	0.50 [0.29, 0.67]	[0.00, 1.00]
	D3 good quality embryo rate	1152	0.18 ± 0.20	0.14 [0.00, 0.28]	[0.00, 1.00]
**Timing Variables**	Trigger to OPU interval, hours	1152	36.81 ± 0.64	36.75 [36.27, 37.25]	[33.97, 39.17]
	OPU to denudation interval, hours	1152	1.66 ± 0.78	1.62 [1.07, 2.15]	[0.17, 6.12]
	Denudation to ICSI interval, hours	1152	2.64 ± 1.04	2.77 [2.05, 3.30]	[0.12, 7.07]

Continuous variables are presented as mean ± standard deviation (SD), median [interquartile range (IQR)], and range [minimum, maximum]. Categorical variables are presented as n (%) with subcategories indented in the N column

Descriptive statistics are calculated for all baseline characteristics. For variables with predominantly zero values (gravidity, parity, abortions, miscarriages, ectopic pregnancies), median and IQR are not displayed. Integer values are used for age variables, infertility duration, basal E2, stimulation duration, gonadotropin dose, E2 at trigger, oocyte counts, and cycle number

Abbreviations: AFC, antral follicle count; AMH, anti-Müllerian hormone; ART, assisted reproductive technology; BMI, body mass index; D2, day 2; D3, day 3; E2, estradiol; FSH, follicle-stimulating hormone; Gn, gonadotropin; ICSI, intracytoplasmic sperm injection; IQR, interquartile range; IU, international units; LH, luteinizing hormone; MII, metaphase II; n, number; OPU, oocyte pickup; 2 PN, two pronuclei; SD, standard deviation; TSH, thyroid-stimulating hormone

The cohort exhibited considerable heterogeneity characteristic of real-world ART populations across demographic profiles, clinical presentations, ovarian reserve markers, and treatment regimens. This diversity included the full spectrum from suboptimal to optimal ovarian responses and conservative to aggressive stimulation strategies, demonstrating individualized clinical decision-making.

### Curvilinear association between DTI and embryo utilization

**Fig. 2:**
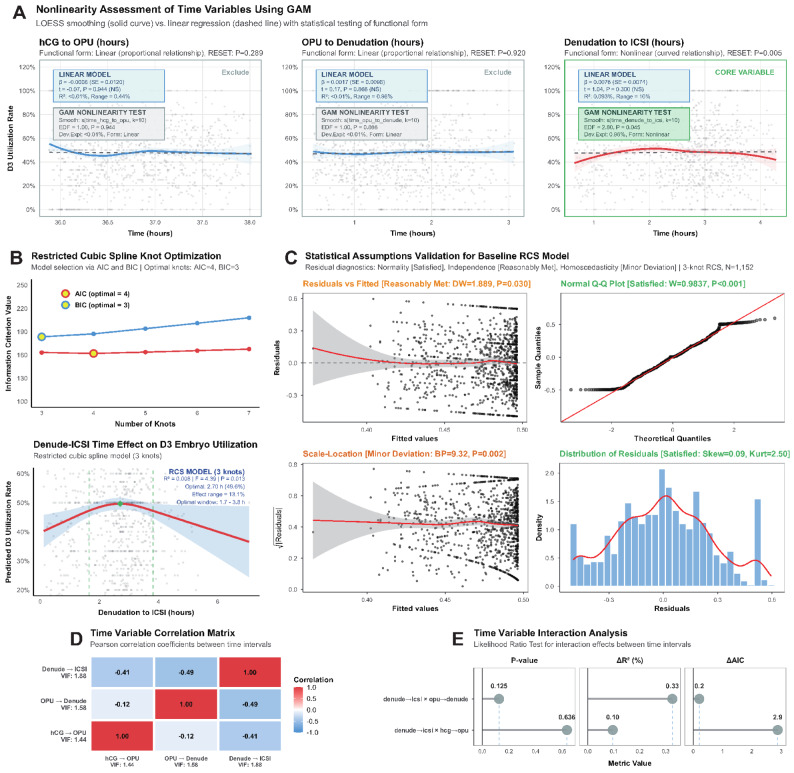
Comprehensive assessment of time variables including nonlinearity tests, independence validation, and RCS optimization. (**A**) Nonlinearity assessment using GAM. Three smooth curves with 95% confidence intervals illustrating relationships between time variables and D3 utilization rate, with DTI demonstrating significant nonlinearity at *p* < 0.05, whereas the other two intervals exhibit linear patterns; (**B**) Restricted cubic spline knot optimization. Two-panel display including RCS prediction curve with optimal timing window and BIC comparison across 3- to 7-knot configurations identifying 3-knot as optimal solution; (**C**) Statistical assumptions validation. Four-panel diagnostic assessment in 2 × 2 layout, including residuals versus fitted values, normal Q-Q plot, scale-location plot, and residuals distribution confirming satisfaction of regression assumptions; (**D**) Time variable relationship matrix. Pearson relationship heatmap among three time intervals with all relationship coefficients below 0.6 and variance inflation factors below 2.0, indicating acceptable independence; (**E**) Time variable interaction analysis. Likelihood ratio test results for two interaction terms between DTI and other time variables exhibiting all *p* > 0.05 and negligible contributions to model fit

Assessment revealed a significant nonlinear association between DTI and day 3 embryo utilization rate, necessitating advanced modeling (Fig. 2). GAM screening revealed significant nonlinearity (EDF = 2.80, *p* < 0.05), whereas linear regression detected no association, confirming the need for nonlinear modeling.

Evaluation of 3–7 knot RCS models identified that the 3-knot specification was optimal, achieving the lowest BIC of 183.3 with knots at 1.00, 2.77, and 3.78 h (10th, 50th, and 90th percentiles). Joint F-test confirmed the significance of the univariate RCS model (F = 4.39, *p* = 0.013), and partial F-test for the nonlinear spline term provided independent evidence of nonlinearity (F = 7.71, *p* = 0.006). Diagnostics revealed adequate assumption adherence, with minor violations deemed insignificant due to the large sample size.

Although model R^2^ was modest, the clinical effect size was substantial. In this unadjusted univariate model, utilization rates fluctuated by 13.1 percentage points across the observed DTI range, peaking at 49.6% at 2.70 h post-denudation, with an acceptable window spanning 1.7–3.8 h. These estimates were subsequently refined through multivariable adjustment and model optimization. Verification confirmed weak-to-moderate correlations among intervals (range: −0.49 to −0.12) with VIF values remaining below thresholds (maximum 1.88) and no interaction effects (all *p* > 0.10).

### Temporal effects confined to embryo development by secondary endpoints

Time interval-outcome evaluation revealed that the DTI effect was confined to embryo utilization rather than earlier endpoints (Figure S1, Table S1). Secondary outcomes were non-significant (day 3 good quality embryo rate: *p* = 0.083; normal fertilization rate: *p* = 0.433; cleavage rate: *p* = 0.364), indicating that the effects of DTI on utilization represent cumulative impacts on development rather than fertilization or cleavage.

The trigger-to-OPU interval exhibited significant nonlinear relationships with fertilization rate (*p* < 0.001, EDF = 2.05) but not day 3 utilization (*p* = 0.249), suggesting effects on oocyte maturation. This parameter represents a well-standardized protocol with minimal variability (36.8 ± 0.64 h), whereas DTI demonstrates substantial operational variability (range: 0.12–7.07 h) and is poorly characterized. Therefore, multivariable modeling concentrated on DTI as the primary modifiable factor affecting day 3 outcomes. The OPU-to-denudation interval revealed non-significant relationships (all *p* > 0.58).

### Association robust to comprehensive covariate adjustment

Model building demonstrated a consistent DTI effect across covariate adjustment levels (Fig.s 3A–F). Variable selection proceeded through three steps: excluding the hCG-to-OPU interval for insufficient variability, VIF-based screening, and univariate testing at *p* < 0.20, yielding 16 variables for multivariable modeling (Tables S2-3).

**Fig. 3:**
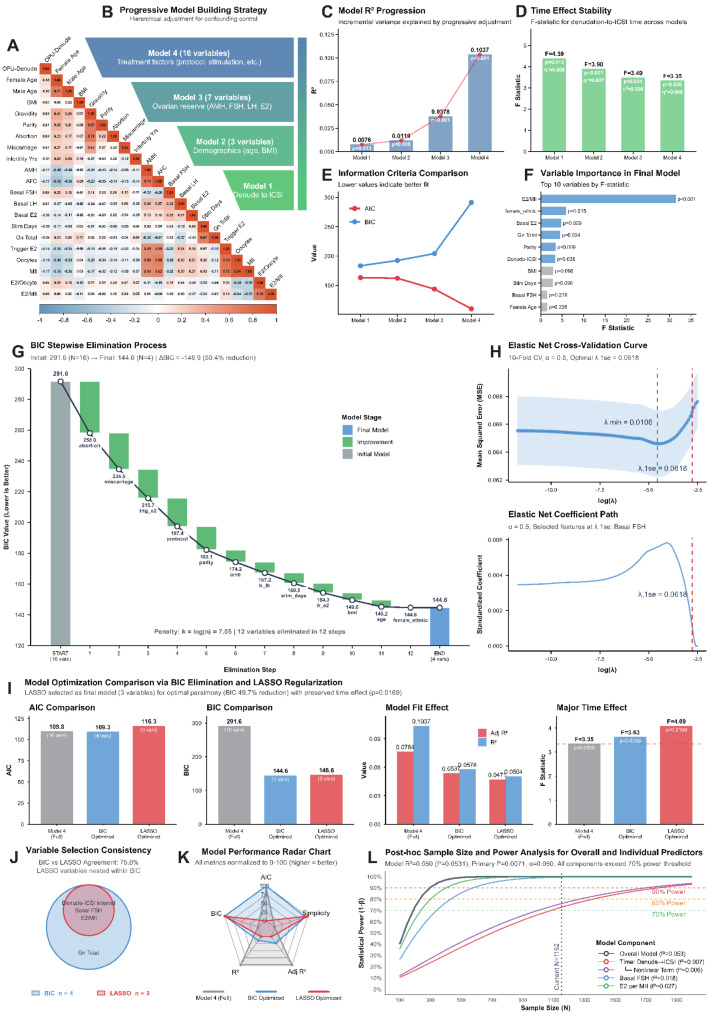
Progressive model building with dual-criteria optimization and post-hoc power analysis. (**A**) Covariate relationship heatmap. Comprehensive relationship matrix of 21 candidate variables before model entry; (**B**) Hierarchical model structure. Four-stage progressive adjustment strategy from univariable to fully adjusted model; (**C**) Model R^2^ progression. Incremental improvement in explained variance across model hierarchy; (**D**) DTI effect stability. Consistency of regression coefficients for DTI throughout model building; (**E**) Information criteria comparison. AIC and BIC values demonstrating progressive model fit improvement; (**F**) Variable importance ranking. Top 10 predictors ordered by F-statistics in Model 4; (**G**) BIC stepwise elimination. Backward selection process reducing variables from 16 to 4 with BIC trajectory; (**H**) Elastic Net cross-validation and coefficient path. Left panel illustrates a 10-fold CV curve at α = 0.5 with optimal lambda, right panel displaying the regularization path with the selected feature at lambda.1se; (**I**) Model performance comparison. Six-metric evaluation of Model 4 versus BIC and LASSO optimized models; (**J**) Variable selection consistency. Venn diagram depicting 75% agreement between BIC and LASSO methods; (**K**) Performance radar chart. Six-dimensional assessment including AIC, BIC, R^2^, adjusted R^2^, and simplicity; (**L**) Post-hoc power analysis. Power curves demonstrating overall model and component-specific power with the current sample size

Hierarchical adjustment yielded consistent results across models (Table S4). The DTI effect remained significant across four progressively adjusted models (Model 1: RCS-transformed DTI alone; Model 2: plus demographics; Model 3: plus ovarian reserve markers; Model 4: plus treatment factors), with a 24% attenuation from the unadjusted to the fully adjusted model, thereby demonstrating robustness to potential confounders. Model performance improved incrementally in parallel, with the largest gains attributable to treatment-related factors, whereas DTI maintained predictive value.

In the fully adjusted model, E2 per MII emerged as the strongest predictor, supplemented by contributions from total gonadotropin dose, ethnicity, and parity. Several variables significant in univariate analysis became non-significant after adjustment, confirming effective confounder control. Model diagnostics confirmed regression assumptions for inference (Figure S2), though modest R^2^ indicates potential for further optimization.

### Parsimonious model achieves superior efficiency through dual-criteria optimization

Dual-criteria optimization identified a parsimonious three-variable model that preserved the essential time effect with improved efficiency (Fig.s 3G–L). BIC-based backward elimination reduced the 16 variables by removing low-contribution predictors (Table S5), whereas elastic net regularization (α = 0.5, λ = 0.062) independently selected comparable variables. Selection consistency reached 75%, exceeding the 70% threshold for intersection-based convergence (Table S6).

The final model retained RCS-transformed DTI, basal FSH, and E2 per MII, achieving an 81% reduction in covariates. The time effect was more pronounced in the parsimonious model (F-statistic: 3.35 to 4.09; *P*: 0.036 to 0.017) (Table 2). DTI exhibited significant curvilinear effects (*p* = 0.017), confirming optimization potential and identifying an optimal timing window. Basal FSH and E2 per MII accounted for ovarian reserve and hormonal environment, with the three-variable model collectively explaining 5.04% of outcome variance. The model satisfied stringent diagnostic standards (Figure S3), including independence and normality of residuals; only 6.6% of observations were influential, and mild heteroscedasticity did not compromise inference validity.

**Table 2:** Regression coefficients and statistical tests of the final optimized model for day 3 embryo utilization rate

Component	Variable	Coefficient (β)	SE	95% CI	t	*p*-value
**Primary Exposure**	DTI (linear term)	0.044	0.016	[0.014, 0.075]	2.84	0.005
	DTI (nonlinear term)	−0.039	0.015	[−0.068, −0.010]	−2.67	0.008
**Covariates**	Basal FSH	0.009	0.002	[0.005, 0.013]	4.60	<0.001
	E2 per MII (change per 100 pg/mL)	−0.005	0.001	[−0.007, −0.003]	−5.52	<0.001
**Model Diagnostics**	Intercept	0.396	0.033	[0.331, 0.461]	11.90	<0.001

Data are presented as Regression coefficients (β), standard errors (SE), 95% confidence intervals (CI), t-statistics, and *p*-values are presented for each model component. The primary exposure (denudation-to-ICSI interval) is decomposed into linear and nonlinear restricted cubic spline terms. Covariates are presented with their respective regression coefficients and statistical tests

The final optimized model is specified to include 3 predictors: (1) denudation-to-ICSI interval (primary exposure), modeled with restricted cubic splines using 3 knots (10th, 50th, 90th percentiles) to capture nonlinear time-dependent effects, generating 2 terms—a linear term for overall trend and a nonlinear term for curvature; (2) basal FSH (IU/L), modeled linearly; (3) E2 per MII (pg/mL), modeled linearly. Coefficients are estimated via ordinary least squares regression. Standard errors are model-based assuming homoscedastic errors. 95% confidence intervals are calculated using t-distribution with degrees of freedom = N - p − 1, where p is the number of predictors including the intercept. The restricted cubic spline transformation uses Harrell’s default knot placement strategy, generating basis functions that partition the predictor space into piecewise cubic polynomials joined smoothly at knots. The linear restricted cubic spline term captures the primary trend, while the nonlinear term captures deviation from linearity

E2 per MII coefficients are rescaled to represent change per 100 pg/mL increase for interpretability. This parsimonious model achieves 81% variable reduction from the full multivariable model (16→3 predictors) while maintaining primary exposure significance (*p* = 0.005) and substantial BIC improvement (ΔBIC = −145.00, 49.7% reduction)

Abbreviations: BIC, Bayesian information criterion; CI, confidence interval; DTI, denudation-to-ICSI interval; E2, estradiol; FSH, follicle-stimulating hormone; ICSI, intracytoplasmic sperm injection; IU, international units; MII, metaphase II oocyte; OLS, ordinary least squares; P, probability; RCS, restricted cubic splines; SE, standard error

### Homogeneous effects across patient strata

Heterogeneity analyses confirmed that DTI effects were consistent across patient characteristics (Fig.s 4A-B, Table S7). Likelihood ratio testing across 14 potential effect modifiers revealed no significant interactions after FDR correction (all adjusted *p* > 0.10), with substantial BIC penalties supporting a unified timing strategy. Stratified analyses across 19 subgroups demonstrated consistent effect direction (89.5%), with significant effects observed among patients with favorable reproductive parameters (Table S8). Cochran Q statistics indicated low-to-moderate heterogeneity across all stratifications (I^2^ < 35%), with negligible between-subgroup variance (τ^2^ < 0.001), suggesting that observed differences were attributable to differential statistical power rather than genuine heterogeneity (Table S9).

**Fig. 4:**
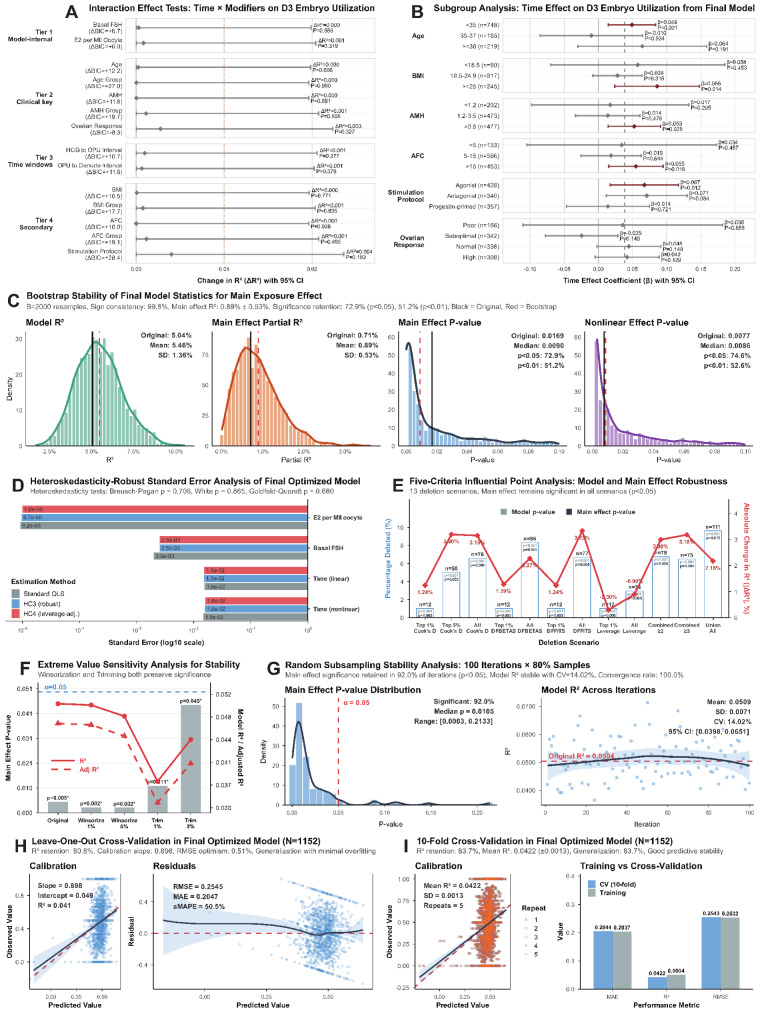
Comprehensive heterogeneity assessment and multi-faceted robustness validation. (**A**) Interaction effect screening. Forest plot of 14 interaction terms between DTI and patient characteristics, with all *p* exceeding 0.05; (**B**) Subgroup analysis. Effect estimates across 19 subgroups with heterogeneity statistics indicating I^2^ below 35%; (**C**) Bootstrap stability analysis. Distribution of 2,000 bootstrap estimates for coefficient, standard error, t-statistic, and *p*; (**D**) Robust standard error comparison. Side-by-side comparison of conventional versus heteroscedasticity-consistent standard errors; (**E**) Influential point sensitivity. Cook’s distance analysis identifying influential observations with deletion diagnostics; (**F**) Extreme value handling. Coefficient stability across untrimmed data and progressive winsorization levels; (**G**) Random subsampling validation. Coefficient and *p*-value distributions from 100 random 80% subsamples; (**H**) Leave-one-out cross-validation. MSE and R^2^ distributions demonstrating prediction stability; (**I**) 10-fold cross-validation. Mean squared error and R^2^ across folds with confidence intervals

Threshold analysis demonstrated that continuous RCS modeling outperformed categorical approaches (tertiles/quartiles), with discrete categorizations substantially penalized by information criteria (Figure S4). These findings support the presence of smooth, continuous time effects without discrete thresholds, confirming mechanistic consistency across patient subgroups and validating the broad applicability of our optimized model for diverse clinical populations.

### High robustness confirmed across multiple analyses

Robustness assessment confirmed the reliability of our findings (Figs. 4C–I, Table S10). Bootstrap resampling (B = 2,000) demonstrated excellent statistical inference stability (99.8% sign consistency), with HC3 robust standard errors maintaining significance despite heteroscedasticity. Sequential removal of influential observations strengthened the DTI effect, suggesting that these points had previously attenuated the true association.

Random subsampling confirmed effect reproducibility in 92% of iterations, and progressive winsorization maintained significance with only moderate coefficient changes. Cross-validation exhibited excellent prediction stability with minimal optimism. Leave-one-out and repeated 10-fold validation retained 80.8% (LOOCV) to 83.7% (10-fold) of training R^2^, confirming good generalization capacity.

### Optimal timing window at 1.75–3.75 h post-denudation

**Fig. 5:**
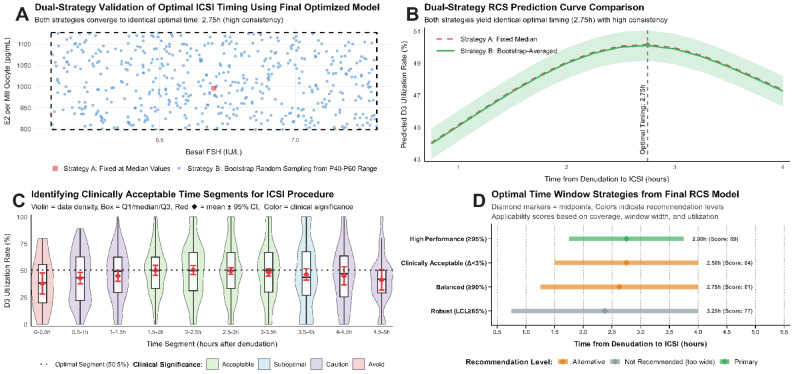
Optimal ICSI timing windows derived from dual-strategy validation with clinical application guidelines. (**A**) Dual-strategy parameter validation. Scatter plot comparing fixed median values versus bootstrap random sampling from P40–P60 range, with both strategies converging to identical optimal timing at 2.75 h; (**B**) RCS prediction curve comparison. Fitted splines from two-parameter strategies demonstrating high consistency in predicted utilization rates across the timing range; (**C**) Time segment analysis. Violin plots with boxplots comparing mean utilization rates across ten half-hourly intervals from 0–0.5 h to 4.5–5 h with color-coded clinical significance categories; (**D**) Window definition strategies. Horizontal timeline illustrating four recommendation levels, including primary high-performance window and alternative balanced options with applicability scores

Two complementary strategies independently identified 2.75 h as optimal timing (Fig. 5). The prediction utilization curve indicated steep improvement before 1.5 h, a gradual plateau to 2.75 h, and a progressive decline beyond 4.0 h. Timing outside 1.5–4.0 h significantly compromised outcomes. Independent 30-min interval analysis demonstrated strong concordance with model predictions. The 2.0–2.5 h segment achieved peak utilization, and adjacent segments exhibited minimal decrements. The earliest segment (<0.5 h) remained significantly inferior, reinforcing the detrimental effect of premature injection.

Using the final optimized model with covariates fixed at median values, predicted utilization rates ranged from 42.9% at DTI of 0.5 h to a peak of 50.2% at 2.75 h, declining to 44.3% at 5.0 h. Within the recommended window of 1.75–3.75 h, predicted rates remained between 48.1% and 50.2%, representing less than 2.1 percentage points of variation. To contextualize these differences, the cohort median oocyte yield was 10 (IQR: 6–16). At optimal timing, a representative cycle would yield approximately 5.0 usable embryos, compared with 4.3 at 0.5 h and 4.4 at 5.0 h, corresponding to 0.6–0.7 additional usable embryos per cycle. At the population level, 30.4% of cycles fell outside the optimal window, with an observed utilization difference of 4.6 percentage points between in-window and out-of-window cycles (49.2% vs. 44.5%), projecting approximately 14 additional usable embryos per 100 cycles if workflow standardization shifted all cycles into the recommended timeframe.

Four window strategies were evaluated to balance optimality with clinical feasibility (Table 3). The high-performance window (1.75–3.75 h) achieved superior composite scoring and was selected as the primary recommendation. This 2 h window covers approximately 70% of patients and maintains near-optimal performance, projected to benefit 30% currently outside this window.

**Table 3:** Optimal ICSI timing windows derived from time segment analysis and restricted cubic spline predictions

Method Category	Strategy Definition	Time Range/ Window	Coverage N (%)	D3 Utilization Rate [95% CI]	vs. Optimal Δ (%)	Clinical Recommendation
**Part A: Time Segment Analysis**	**Early Segments**	0–0.5 h	25 (2.2%)	37.9% [28.6, 47.3]	−12.6%	Avoid
		0.5-1 h	83 (7.3%)	43.0% [37.8, 48.1]	−7.5%	Caution
		1–1.5 h	80 (7.0%)	45.1% [40.0, 50.2]	−5.4%	Caution
	**Optimal Segments**	1.5-2 h	85 (7.5%)	50.1% [45.5, 54.8]	−0.4%	Acceptable
		2–2.5 h	153 (13.4%)	50.5% [46.4, 54.6]	+0.0%	Acceptable
		2.5-3 h	285 (25.0%)	49.6% [46.6, 52.6]	−1.0%	Acceptable
		3–3.5 h	229 (20.1%)	48.2% [44.9, 51.6]	−2.3%	Acceptable
	**Late Segments**	3.5-4 h	131 (11.5%)	46.5% [41.2, 51.7]	−4.0%	Suboptimal
		4–4.5 h	42 (3.7%)	45.1% [36.8, 53.4]	−5.5%	Caution
		4.5-5 h	26 (2.3%)	41.3% [32.2, 50.4]	−9.2%	Caution
**Part B: RCS Window Strategies**	**High Performance** **(≥95%)**	1.75 - 3.75 h (Width: 2.00 h)	802 (69.6%)	49.2% (RCS Predicted)	−1.3%	Primary (Score: 89.4)
	**Clinically Acceptable** **(Δ < 3%)**	1.50 - 4.00 h (Width: 2.50 h)	885 (76.8%)	48.9% (RCS Predicted)	−1.6%	Alternative (Score: 84.4)
	**Balanced** **(≥90%)**	1.25 - 4.00 h (Width: 2.75 h)	917 (79.6%)	48.6% (RCS Predicted)	−1.9%	Alternative (Score: 81.2)
	**Robust** **(LCL ≥ 85%)**	0.75 - 4.00 h (Width: 3.25 h)	1017 (88.3%)	48.1% (RCS Predicted)	−2.5%	Not Recommended (Score: 77.2)

Data are presented as time ranges (hours), sample coverage (n and %), D3 embryo utilization rates (% with 95% confidence intervals), and differences from optimal (Δ, %). Clinical recommendation levels are assigned as Primary, Alternative, Not Recommended, Acceptable, Suboptimal, Caution, or Avoid

Part A presents time segment analysis using observed data from 0.5-hour discrete intervals. Segments with n ≥ 20 are retained and grouped as Early, Optimal, and Late based on clinical significance criteria (Acceptable: |Δ|<3%, Suboptimal: 3–5%, Caution: 5–10%, Avoid: ≥10%). The segment with highest mean utilization serves as reference for calculating differences. Part B presents RCS window strategies using continuous prediction-based windows derived from restricted cubic spline model with 3 knots, adjusted for basal FSH and E2 per MII oocyte at median values. Four strategies are defined by performance thresholds relative to maximum predicted utilization: High Performance (≥95%), Balanced (≥90%), Robust (lower confidence limit ≥ 85%), and Clinically Acceptable (absolute decline < 3%). Applicability scores are calculated as weighted composite of efficiency (40%), coverage (30%), precision (20%), and clinical feasibility (10%). For Part B strategies, Primary recommendation indicates optimal balance of coverage (60–80%) and precision (width 0.5–2.0 h); Alternative recommendation indicates acceptable tradeoff between coverage and precision; Not Recommended indicates excessively wide windows (>3 h) or insufficient coverage (<40%)

Abbreviations: CI, confidence interval; D3, day 3; E2, estradiol; FSH, follicle-stimulating hormone; LCL, lower confidence limit; MII, metaphase II; RCS, restricted cubic spline; Δ, difference

## Discussion

The findings revealed a robust nonlinear association between DTI and day 3 embryo utilization rate, identifying an operational window of 1.75–3.75 h associated with clinically meaningful differences in predicted outcomes. This finding addresses a longstanding translational gap in reproductive medicine. Despite decades of ICSI practice, evidence-based timing guidelines have remained elusive, leading laboratories to rely on conventional methods rather than optimized protocols. The timing effect exhibited consistency across all 19 patient subgroups without significant interactions, indicating a biological phenomenon amenable to standardized implementation rather than necessitating patient-specific modifications.

Despite extensive investigation, the optimal ICSI timing remains debatable. The overall hCG-to-ICSI interval of 37–41 h has been reported as optimal for implantation [25]; however, recent systematic reviews have predominantly concentrated on laboratory-specific OPU-to-ICSI intervals rather than the DTI window examined here [13, 26]. The significant heterogeneity across existing studies, encompassing diverse interval definitions, inconsistent confounder adjustment, and absence of nonlinearity assessment, highlights the need for methodologically rigorous investigations.

Our findings align with studies demonstrating that DTI independently predicts reproductive outcomes. One study indicated enhanced outcomes with longer intervals [27], another identified optimal outcomes within 4 h post-denudation [28], and a third examined OPU-ICSI and denudation-ICSI intervals, demonstrating that combining adequate pre- and post-denudation timing maximized fertilization rates [29]. Our study extends these observations through several methodological refinements. Continuous nonlinear modeling employing RCS captured the underlying dose-response relationship without imposing predetermined categorical thresholds, revealing nuanced patterns that dichotomous analyses tend to obscure. Day 3 embryo utilization rate was selected as the primary endpoint to capture the full spectrum of laboratory process efficiency from retrieval through culture. International consensus frameworks have recognized embryo utilization as a relevant performance concept, defining it as embryos transferred or cryopreserved relative to normally fertilized oocytes [3]. In our study, the numerator comprised grade A, B, and C embryos, all of which met institutional criteria for transfer or cryopreservation, thereby representing the same conceptual pool of clinically usable embryos while being determined by objective morphological assessment rather than variable clinical decisions. The denominator was set at oocytes retrieved rather than fertilized oocytes, because the exposure under investigation occurs before fertilization and its cumulative effects on both fertilization and subsequent development needed to be captured. Day 3 assessment further represents the universal embryo disposition decision point at which virtually all laboratories determine embryo fate, regardless of whether subsequent protocols involve fresh transfer, extended culture, or cryopreservation. Systematic multivariable adjustment, an aspect often inadequately addressed in prior work, was implemented through VIF-based screening to a parsimonious model, complemented by extensive heterogeneity analysis confirming effect consistency across patient subgroups.

Discordant findings in the literature necessitate interpretation across several dimensions. Previous research examined conceptually different exposure windows. Some investigated trigger-to-OPU intervals with protocol-dependent effects [30], others included trigger-to-injection intervals encompassing both in vivo maturation and laboratory handling phases [31], and still others assessed broader OPU-to-ICSI windows without differentiating between pre- and post-denudation components [32], or composite timing segments without specifically isolating the DTI interval [33]. Variations in denudation techniques, especially those involving immediate ICSI following denudation [34] or extended pre-denudation incubation [35], further limited assessment of the specific DTI window. Categorical grouping with established thresholds may have further diminished the ability to identify nonlinear dose-response relationships. Internal validation within our dataset demonstrated that categorical approaches failed to detect timing effects even in subgroups where continuous RCS modeling revealed significant associations, with all categorical specifications yielding non-significant results and substantial information criterion penalties (Figure S4). Although age-stratified analyses indicate that timing sensitivity may differ among populations [36], our comprehensive subgroup analyses detected no significant effect modification across 19 strata, supporting the broader applicability of the identified optimal window. The DTI effect, characterized by a small partial f^2^ of 0.007, required both flexible nonlinear modeling and substantial sample sizes for reliable detection, which may explain prior null findings in studies employing categorical groupings in smaller cohorts.

The denudation procedure establishes a critical vulnerability window by removing the cumulus cell layer, which facilitates essential metabolic cooperation through gap junctions [37], antioxidant defenses via glutathione and catalase [38], and paracrine signaling through EGF-like peptides, including amphiregulin and epiregulin [39]. After cumulus removal, oocytes depend exclusively on zona pellucida protection, making them highly susceptible to micromanipulation timing. Our observed inverted U-shaped relationship, indicating optimal utilization at 2.75 h, implies that oocyte quality undergoes dynamic temporal variations reflecting underlying molecular transitions rather than acute fertilization events.

We propose a triphasic temporal model to explain these time-dependent quality changes. During the initial 0–2 h following cumulus removal, an acute stress response predominates, driven by hyaluronidase-induced cellular stress [40], loss of metabolic support with decreased ATP levels [41, 42], and possible polar body displacement from mechanical manipulation [43]. Between 2 and 4 h, stress recovery appears complete and the oocyte enters an optimal maturation state, with nuclear-cytoplasmic synchronization [44], restored mitochondrial activity [45], reestablished oxidative homeostasis [46], and maintained responsiveness to sperm-induced calcium oscillations [47]. Beyond 4 h, rapid aging ensues in the absence of cumulus protection, as advancing mitochondrial dysfunction [48], oxidative damage accumulation [49], calcium homeostasis disturbances [50], and spindle abnormalities [51] progressively compromise oocyte competence. Denuded oocytes age more rapidly than cumulus-enclosed oocytes [52]. The lack of immediate effects on fertilization rate but significant impact on day 3 embryo utilization substantiates a cumulative developmental hypothesis in which subtle molecular perturbations during the peri-denudation period accumulate through successive cell divisions, manifesting as divergent embryo quality at the cleavage stage.

The R^2^ of 5.04% warrants contextualization within the multifactorial nature of embryo development. Oocyte developmental competence is governed by genetic constitution, sperm DNA integrity, culture conditions, and numerous factors not modifiable at the point of injection. In this context, a single procedural parameter explaining approximately 5% of outcome variance represents a meaningful and actionable target, particularly given its complete modifiability at no marginal cost. The theoretical ceiling for any single timing variable is inherently constrained when dozens of upstream determinants, most of which are fixed at the point of injection, collectively govern the outcome. Absolute metrics offer a more intuitive measure of practical impact. The 4.6 percentage-point observed difference between in-window and out-of-window cycles translates to approximately 14 additional usable embryos per 100 cycles treated, a gain achievable solely through workflow scheduling without additional reagents, equipment, or patient burden. A low R^2^ indicates limited capacity to predict outcomes for individual cycles, a constraint inherent to any single-variable optimization in a multifactorial biological system. The clinical relevance instead derives from the reliable identification of a modifiable parameter with consistent population-level effects, as confirmed by bootstrap resampling and cross-validation analyses.

A critical advantage of our findings lies in their immediate translational potential for laboratory workflow optimization. Unlike time-lapse monitoring systems that necessitate significant capital investment [53], implementation of our findings entails only scheduling adjustments within existing workflows. Effective adoption would still require workflow reorganization, staff training, and scheduling coordination, the feasibility and scope of which may vary across centers. The core recommendation of targeting the 1.75–3.75 h window while avoiding ICSI within 1.5 h post-denudation affords sufficient flexibility for standard laboratory practice without requiring stringent adherence to a narrow time point. The absence of significant interaction effects across 19 patient subgroups suggests broad applicability without the need for individualized timing modifications, significantly simplifying clinical implementation.

This resource-independent characteristic facilitates adoption across all center sizes and healthcare settings, from well-funded academic institutions to smaller clinics in resource-limited settings. Approximately 30% of cycles conducted outside the optimal window represent a significant opportunity for enhancing outcomes solely through workflow standardization. From a health economics perspective, timing optimization offers potential benefit without incremental cost and is applicable at scale without infrastructure requirements.

Our findings also address persistent gaps in international guidelines. The 2015 ESHRE guidance and 2022 ASRM recommendations acknowledge insufficient evidence for specific DTI parameters [15, 54], indicating only temporal proximity without quantitative thresholds. By supplying statistical evidence for defined optimal windows with specific threshold recommendations, we offer quantitative guidance that may be directly integrated into laboratory standard operating procedures, representing a concrete step toward evidence-based standardization of ICSI practice.

This study adhered to STROBE guidelines to ensure methodological transparency [55]. The real-world design using routine clinical data offers external validity through representative populations and realistic operational conditions [56]. Several technical features enhance reliability. Complete variable capture minimizes selection bias, and minute-level time precision prevents categorical threshold artifacts [57]. Prospective time-stamping through mandatory patient identity verification mitigates recall bias and precludes retrospective modification, with any residual imprecision representing non-differential measurement error that would bias estimates toward the null. Standardized embryo grading ensures outcome consistency. The single-center design further ensures uniform protocols, consistent equipment, and standardized operator training, minimizing procedural heterogeneity that could obscure the DTI-outcome relationship. Analytically, our GAM-to-RCS approach systematically merges exploratory flexibility with confirmatory precision [58], whereas dual-criteria variable selection integrating stepwise elimination and least absolute shrinkage and selection operator (LASSO) regularization improves predictor identification robustness [59]. Comprehensive validation across bootstrap stability, influential point sensitivity, extreme value analysis, cross-validation, heterogeneity testing, and interaction screening provides converging evidence for result reliability.

Despite these strengths, several limitations warrant consideration. As an observational study, our design can establish association but not causation, although the observed relationships are consistent with multiple Bradford Hill criteria, including strength of association, consistency across subgroups, biological gradient, and plausibility [60]. Definitive causal inference awaits confirmation through prospective randomized trials. The single-center design limits immediate generalizability to populations with different ethnic compositions or alternative laboratory platforms. The biological processes underlying the DTI effect, primarily cytoskeletal recovery and spindle reassembly, are governed by conserved cellular mechanisms, and no significant heterogeneity was detected across demographic subgroups within our multiethnic cohort. External validation in centers with different laboratory configurations, culture systems, and patient populations remains warranted before the identified timing window can be recommended for widespread implementation. Individual embryologist identity, denudation batch size, and incubator allocation were not recorded and could not be included as covariates or random effects. Although day 3 utilization offers a clinically relevant and standardized endpoint, we lack data on pregnancy and live birth outcomes, representing a significant limitation for definitive clinical translation. The relationship between improved embryo availability and cumulative live birth rates may be modulated by embryo selection strategies, transfer policies, endometrial receptivity, and patient-specific factors beyond the scope of laboratory timing optimization. Future priorities should include multicenter randomized trials to validate optimal timing windows using cumulative live birth rates as the primary endpoint, mechanistic investigations to elucidate the biological basis of the timing effect, and development of prediction models that might identify patient subgroups with differential timing sensitivity not captured in our heterogeneity analyses.

## Conclusions

This cohort study revealed a robust nonlinear association between DTI and day 3 embryo utilization, identifying a window at 1.75–3.75 h post-denudation within which predicted utilization rates were highest. This time-based optimization requires no additional equipment or resources and has the potential to increase embryo availability for substantial patient populations through procedural standardization alone. Given the observational nature of this study, prospective randomized trials remain necessary to confirm these findings and establish causal relationships.

## Electronic supplementary material

Below is the link to the electronic supplementary material.

Supplementary Figure 1

Supplementary Figure 2

Supplementary Figure 3

Supplementary Figure 4

Supplementary Table 1

Supplementary Table 2

Supplementary Table 3

Supplementary Table 4

Supplementary Table 5

Supplementary Table 6

Supplementary Table 7

Supplementary Table 8

Supplementary Table 9

Supplementary Table 10

## Data Availability

The datasets used and/or analyzed during the current study are available from the corresponding author on reasonable request, subject to institutional review board approval and data use agreements to protect patient privacy.

## Abbreviations

AIC: Akaike information criterion
AMH: Anti-Müllerian hormone
ART: Assisted reproductive technology
ASRM: American Society for Reproductive Medicine
ATP: Adenosine triphosphate
BIC: Bayesian information criterion
CI: Confidence interval
COC: Cumulus-oocyte complex
CV: Cross-validation
DNA: Deoxyribonucleic acid
DTI: Denudation-to-ICSI interval
EDF: Effective degrees of freedom
EGF: Epidermal growth factor
ESHRE: European Society of Human Reproduction and Embryology
E2: Estradiol
FSH: Follicle-stimulating hormone
GAM: Generalized additive model
GnRH: Gonadotropin-releasing hormone
hCG: Human chorionic gonadotropin
HC3: Heteroscedasticity-consistent standard errors (type 3)
ICSI: Intracytoplasmic sperm injection
IQR: Interquartile range
IVF: In vitro fertilization
LASSO: Least absolute shrinkage and selection operator
LOOCV: Leave-one-out cross-validation
MAE: Mean absolute error
MII: Metaphase II
OPU: Oocyte pickup
RCS: Restricted cubic spline
RMSE: Root mean square error
SD: Standard deviation
STROBE: Strengthening the Reporting of Observational Studies in Epidemiology
VIF: Variance inflation factor
