# Supplementary material for: Nonlinear effects of post-denudation timing on day 3 embryo outcomes in ICSI and evidence for a translatable optimization window
Source: J Transl Med. 2026 Jul 11;24:894. doi: 10.1186/s12967-026-08586-0 (PMC13366850; doi:10.1186/s12967-026-08586-0)
Supplement: Supplementary file 1 — Supplementary Figure 1 [file 12967_2026_8586_MOESM1_ESM.pdf]

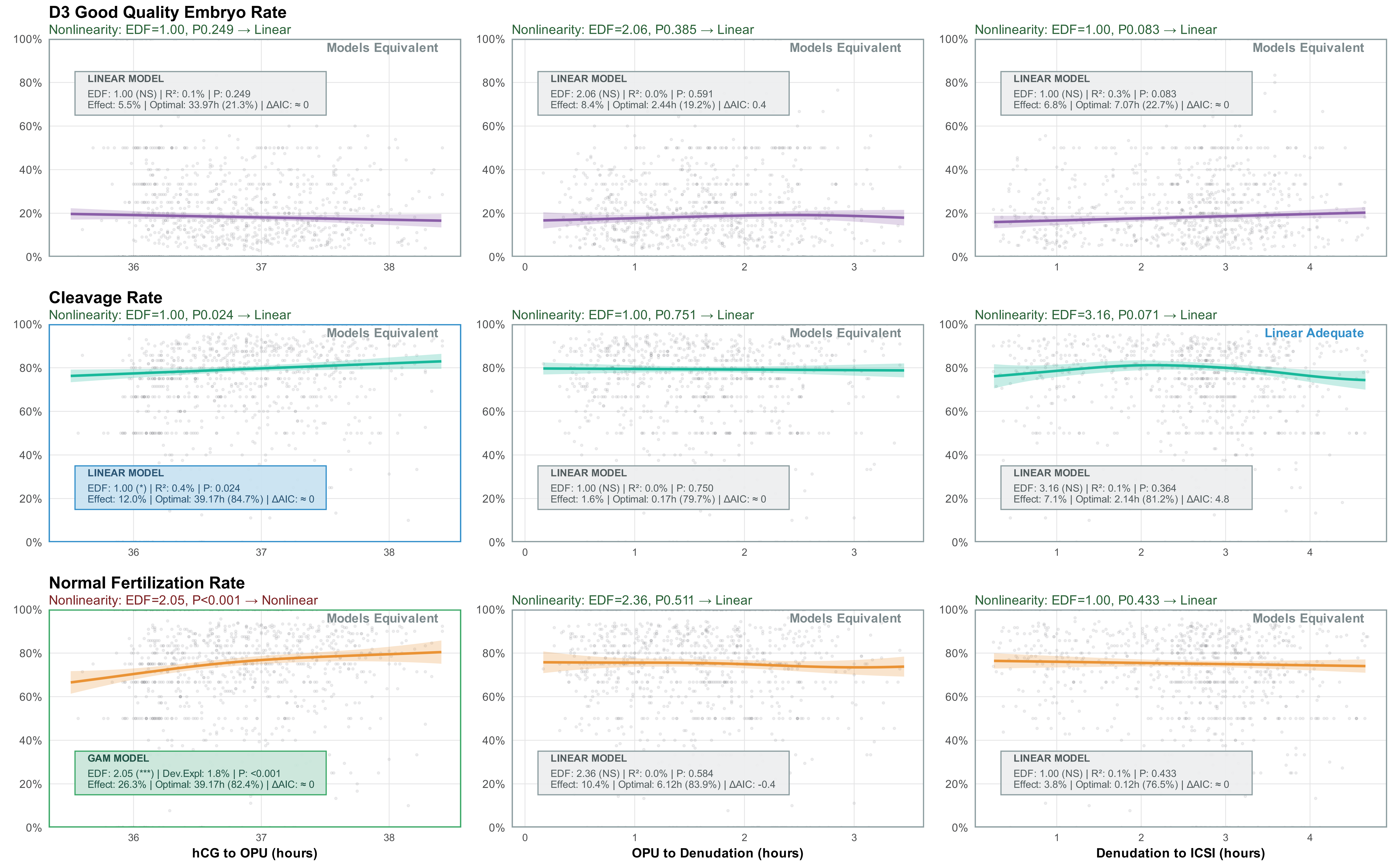

D3 Good Quality Embryo Rate
Nonlinearity: EDF=1.00, P0.249 → Linear
Models Equivalent
LINEAR MODEL
EDF: 1.00 (NS) | R²: 0.1% | P: 0.249
Effect: 5.5% | Optimal: 33.97h (21.3%) | ΔAIC: ≈ 0
Nonlinearity: EDF=2.06, P0.385 → Linear
Models Equivalent
LINEAR MODEL
EDF: 2.06 (NS) | R²: 0.0% | P: 0.591
Effect: 8.4% | Optimal: 2.44h (19.2%) | ΔAIC: 0.4
Nonlinearity: EDF=1.00, P0.083 → Linear
Models Equivalent
LINEAR MODEL
EDF: 1.00 (NS) | R²: 0.3% | P: 0.083
Effect: 6.8% | Optimal: 7.07h (22.7%) | ΔAIC: ≈ 0
Cleavage Rate
Nonlinearity: EDF=1.00, P0.024 → Linear
Models Equivalent
LINEAR MODEL
EDF: 1.00 (*) | R²: 0.4% | P: 0.024
Effect: 12.0% | Optimal: 39.17h (84.7%) | ΔAIC: ≈ 0
Nonlinearity: EDF=1.00, P0.751 → Linear
Models Equivalent
LINEAR MODEL
EDF: 1.00 (NS) | R²: 0.0% | P: 0.750
Effect: 1.6% | Optimal: 0.17h (79.7%) | ΔAIC: ≈ 0
Nonlinearity: EDF=3.16, P0.071 → Linear
Linear Adequate
LINEAR MODEL
EDF: 3.16 (NS) | R²: 0.1% | P: 0.364
Effect: 7.1% | Optimal: 2.14h (81.2%) | ΔAIC: 4.8
Normal Fertilization Rate
Nonlinearity: EDF=2.05, P<0.001 → Nonlinear
Models Equivalent
GAM MODEL
EDF: 2.05 (***) | Dev.Expl: 1.8% | P: <0.001
Effect: 26.3% | Optimal: 39.17h (82.4%) | ΔAIC: ≈ 0
Nonlinearity: EDF=2.36, P0.511 → Linear
Models Equivalent
LINEAR MODEL
EDF: 2.36 (NS) | R²: 0.0% | P: 0.584
Effect: 10.4% | Optimal: 6.12h (83.9%) | ΔAIC: -0.4
Nonlinearity: EDF=1.00, P0.433 → Linear
Models Equivalent
LINEAR MODEL
EDF: 1.00 (NS) | R²: 0.1% | P: 0.433
Effect: 3.8% | Optimal: 0.12h (76.5%) | ΔAIC: ≈ 0
0%
20%
40%
60%
80%
100%
36
37
38
0
1
2
3
4
hCG to OPU (hours)
OPU to Denudation (hours)
Denudation to ICSI (hours)
