# Supplementary material for: Nonlinear effects of post-denudation timing on day 3 embryo outcomes in ICSI and evidence for a translatable optimization window
Source: J Transl Med. 2026 Jul 11;24:894. doi: 10.1186/s12967-026-08586-0 (PMC13366850; doi:10.1186/s12967-026-08586-0)

## Model Diagnostic Plots for Final Optimized Model

Residual diagnostics: Normality [Satisfied], Independence [Satisfied], Homoscedasticity [Minor Deviation] | N=1,152

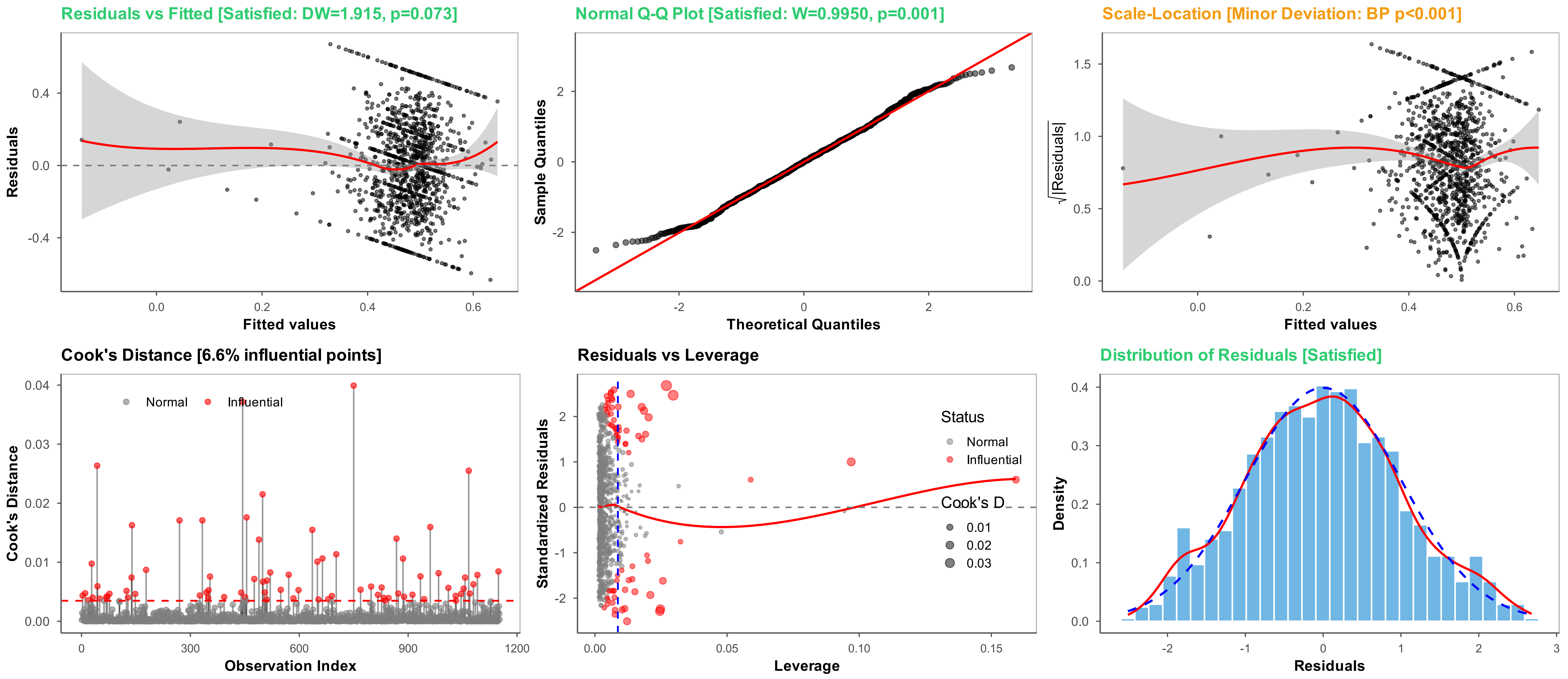

Supplement: Supplementary file 3 — Supplementary Figure 3 [file 12967_2026_8586_MOESM3_ESM.pdf]
