# Supplementary material for: Nonlinear effects of post-denudation timing on day 3 embryo outcomes in ICSI and evidence for a translatable optimization window
Source: J Transl Med. 2026 Jul 11;24:894. doi: 10.1186/s12967-026-08586-0 (PMC13366850; doi:10.1186/s12967-026-08586-0)

Threshold Analysis in Final Optimized Model (n=1152): Stratified RCS Curves and Quantile-Based Segmentation Across 5 Significant Subgroups

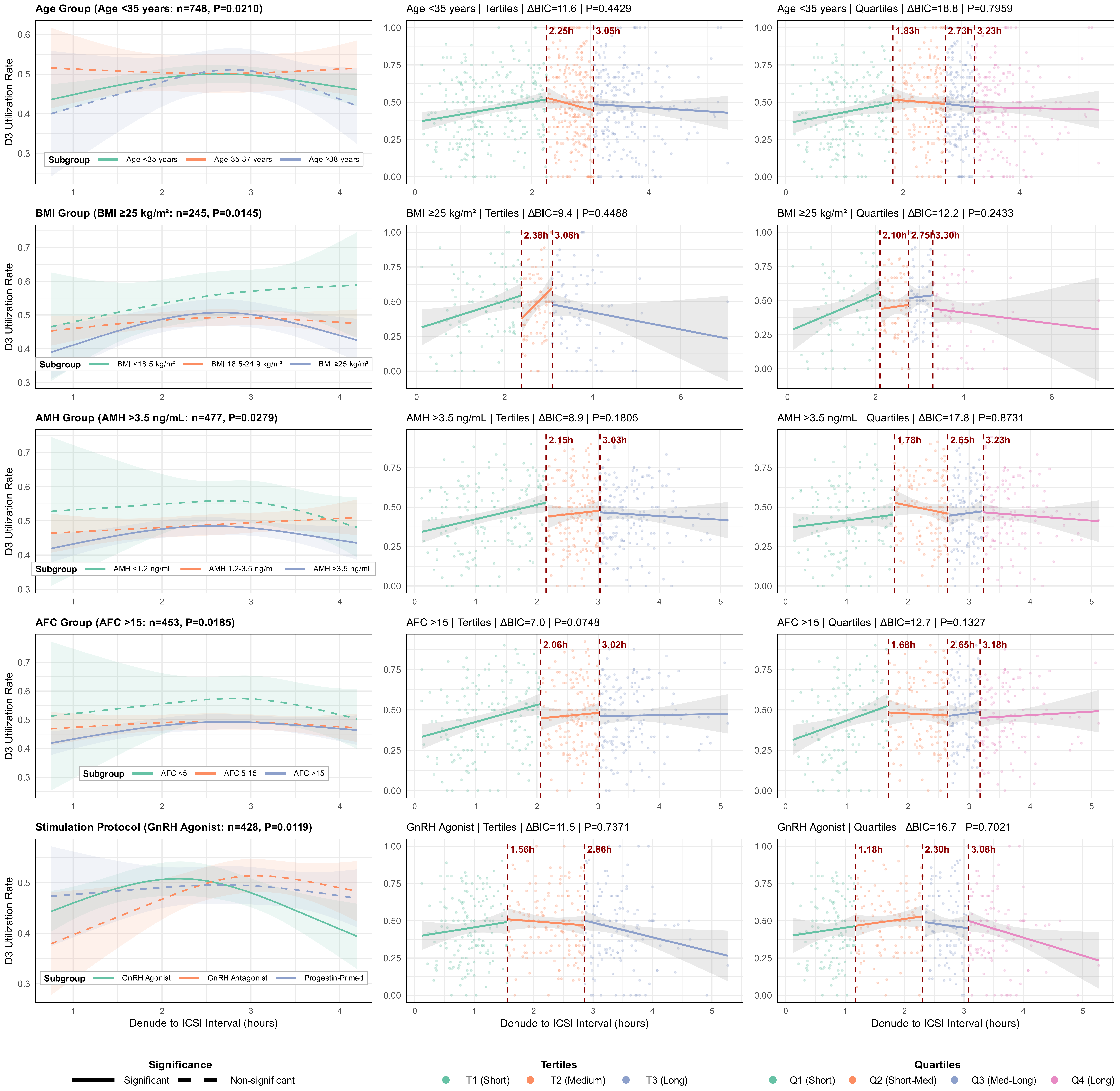

Supplement: Supplementary file 4 — Supplementary Figure 4 [file 12967_2026_8586_MOESM4_ESM.pdf]
