# Supplementary material for: Nonlinear effects of post-denudation timing on day 3 embryo outcomes in ICSI and evidence for a translatable optimization window
Source: J Transl Med. 2026 Jul 11;24:894. doi: 10.1186/s12967-026-08586-0 (PMC13366850; doi:10.1186/s12967-026-08586-0)
Supplement: Supplementary file 5 — Supplementary Table 1 [file 12967_2026_8586_MOESM5_ESM.docx]

**Table S1. Associations between three time interval variables and secondary laboratory outcomes using generalized additive models with restricted cubic spline validation**

| **Timing Variable** | **Outcome** | **N** | **Relationship Form** | **Effect (%)** | **Overall P-value** | **Nonlinearity P-value** | **R² (%)** | **Optimal Point (Time & Value)** | **Model Selection (ΔAIC)** | **RCS Validation (Consistency)** |
| --- | --- | --- | --- | --- | --- | --- | --- | --- | --- | --- |
| **Trigger to OPU** | Fertilization Rate | 1,152 | Nonlinear (EDF=2.05) | 26.3 | <0.001 | <0.001 | 1.8 | 39.17h (82.4%) | Equiv (ΔAIC=0.0) | Moderate (Δ=9.1%) |
|  | Cleavage Rate | 1,152 | Linear (EDF=1.00) | 12.0 | 0.0240 | 0.0240 | 0.4 | 39.17h (84.7%) | Equiv (ΔAIC=-0.0) | — |
|  | Good Embryo Rate | 1,152 | Linear (EDF=1.00) | 5.5 | 0.2487 | 0.2490 | 0.1 | 33.97h (21.3%) | Equiv (ΔAIC=-0.0) | — |
| **OPU to Denudation** | Fertilization Rate | 1,152 | Linear (EDF=2.36) | 10.4 | 0.5842 | 0.5112 | 0.3 | 6.12h (83.9%) | Equiv (ΔAIC=-0.4) | — |
|  | Cleavage Rate | 1,152 | Linear (EDF=1.00) | 1.6 | 0.7503 | 0.7505 | 0.0 | 0.17h (79.7%) | Equiv (ΔAIC=-0.0) | — |
|  | Good Embryo Rate | 1,152 | Linear (EDF=2.06) | 8.4 | 0.5906 | 0.3847 | 0.3 | 2.44h (19.2%) | Equiv (ΔAIC=0.4) | — |
| **Denudation to ICSI** | Fertilization Rate | 1,152 | Linear (EDF=1.00) | 3.8 | 0.4328 | 0.4328 | 0.1 | 0.12h (76.5%) | Equiv (ΔAIC=-0.0) | — |
|  | Cleavage Rate | 1,152 | Linear (EDF=3.16) | 7.1 | 0.3639 | 0.0708 | 1.0 | 2.14h (81.2%) | Linear (ΔAIC=4.8) | — |
|  | Good Embryo Rate | 1,152 | Linear (EDF=1.00) | 6.8 | 0.0834 | 0.0835 | 0.3 | 7.07h (22.7%) | Equiv (ΔAIC=-0.0) | — |
| *Data are presented as relationship form (linear or nonlinear determined by effective degrees of freedom), effect size (%), overall P-value, nonlinearity P-value, R², optimal time point with corresponding outcome value, model selection classification, and RCS validation consistency rating. Effect (%) represents the predicted outcome range (maximum minus minimum) across the observed time range (5th to 95th percentile). Overall P-value tests whether the timing variable has a significant association with the outcome. Nonlinearity P-value specifically tests whether the smooth term differs significantly from a linear relationship (values close to 1.0 indicate linear pattern; values <0.05 combined with EDF >1.5 confirm significant nonlinearity). R² represents deviance explained by GAM (analogous to R² in linear regression). Optimal point indicates the time yielding maximum predicted outcome with corresponding value.* | | | | | | | | | | |
| *Generalized additive models (GAM) with thin plate regression spline (k=10) are fitted using REML estimation. Nonlinearity is assessed by effective degrees of freedom (EDF >1.5) and Wald test P-value. Model selection is based on Akaike Information Criterion difference (ΔAIC = Linear_AIC − GAM_AIC), classified as: Strong GAM (ΔAIC <−10, strong evidence for GAM), GAM (ΔAIC <−2, moderate evidence), Equiv (\|ΔAIC\| <2, models equivalent), or Linear (ΔAIC >2, linear model sufficient). For significant nonlinear models (P <0.05, EDF >1.5), restricted cubic spline validation is performed with optimal knot number derived from GAM EDF. RCS validation consistency assesses agreement between GAM and RCS approaches: Excellent (effect difference <2% AND time difference <0.5h), Good (effect <5% AND time <1h), Moderate (otherwise).* | | | | | | | | | | |
| *RCS validation consistency is displayed only for significant nonlinear models; otherwise shown as —.* | | | | | | | | | | |
| *Abbreviations: AIC, Akaike Information Criterion; EDF, effective degrees of freedom; GAM, generalized additive model; hCG, human chorionic gonadotropin; ICSI, intracytoplasmic sperm injection; OPU, oocyte pick-up; RCS, restricted cubic spline; REML, restricted maximum likelihood; ΔAIC, AIC difference; —, not applicable.* | | | | | | | | | | |
