# Supplementary material for: Nonlinear effects of post-denudation timing on day 3 embryo outcomes in ICSI and evidence for a translatable optimization window
Source: J Transl Med. 2026 Jul 11;24:894. doi: 10.1186/s12967-026-08586-0 (PMC13366850; doi:10.1186/s12967-026-08586-0)
Supplement: Supplementary file 6 — Supplementary Table 2 [file 12967_2026_8586_MOESM6_ESM.docx]

**Table S2. Multicollinearity diagnostics before and after VIF-based variable exclusion**

| **Category** | **Variable** | **Initial VIF** | **Correlated With (\|r\|>0.7)** | **r*** | **Decision** | **Exclusion Reason** | **Post-Exclusion VIF** | **Final Status** |
| --- | --- | --- | --- | --- | --- | --- | --- | --- |
| **Time Intervals** | OPU to denudation interval | 1.64 | — | — | **Retained** | — | 1.60 | Passed |
|  | Denudation to ICSI interval | 1.76 | — | — | **Retained** | — | 1.69 | Passed |
| **Demographics** | Female age | 2.67 | Male age | 0.708 | **Retained** | — | 1.61 | Passed |
|  | Male age | 2.07 | Female age | 0.708 | **Excluded** | Correlated with female age (r=0.708); retained female age (clinical priority) | — | Excluded |
|  | Female BMI | 1.18 | — | — | **Retained** | — | 1.10 | Passed |
| **Reproductive History** | Gravidity | 13.56 | Abortions | 0.705 | **Excluded** | High VIF (13.56) + correlated with abortions (r=0.705) | — | Excluded |
|  | Parity | 4.63 | — | — | **Retained** | — | 1.17 | Passed |
|  | Abortions | 5.59 | Gravidity | 0.705 | **Retained** | — | 1.11 | Passed |
|  | Miscarriages | 3.00 | — | — | **Retained** | — | 1.10 | Passed |
|  | Infertility duration | 1.18 | — | — | **Retained** | — | 1.13 | Passed |
| **Ovarian Reserve** | AMH | 2.61 | AFC | 0.732 | **Retained** | — | 1.98 | Passed |
|  | AFC | 3.04 | AMH | 0.732 | **Excluded** | Correlated with AMH (r=0.732); retained AMH (clinical priority) | — | Excluded |
|  | Basal FSH | 1.53 | — | — | **Retained** | — | 1.50 | Passed |
|  | Basal LH | 1.47 | — | — | **Retained** | — | 1.44 | Passed |
|  | Basal E2 | 1.22 | — | — | **Retained** | — | 1.21 | Passed |
| **Stimulation Response** | Stimulation duration | 2.26 | — | — | **Retained** | — | 2.25 | Passed |
|  | Total Gn dose | 2.48 | — | — | **Retained** | — | 2.44 | Passed |
|  | E2 at trigger | 4.24 | Oocytes retrieved | 0.756 | **Retained** | — | 1.81 | Passed |
| **Oocyte Yield** | Oocytes retrieved | 13.32 | MII oocytes | 0.937 | **Excluded** | High VIF (13.32) + correlated with MII oocytes (r=0.937) | — | Excluded |
|  | MII oocytes | 11.58 | Oocytes retrieved | 0.937 | **Excluded** | High VIF (11.58) + correlated with trigger E2 (r=0.722) | — | Excluded |
| **Normalized Metrics** | E2 per oocyte | 3.09 | E2 per MII | 0.705 | **Excluded** | Correlated with E2 per MII (r=0.705); retained E2/MII (more stable) | — | Excluded |
|  | E2 per MII | 2.92 | E2 per oocyte | 0.705 | **Retained** | — | 1.09 | Passed |
| *Data are presented as Initial VIF values, correlated variables (\|r\|>0.7), correlation coefficients (r), exclusion decisions with rationale, and post-exclusion VIF values are presented for all candidate predictors evaluated for multicollinearity.* | | | | | | | | |
| *VIF is calculated as 1/(1-R²), where R² represents the coefficient of determination when regressing each predictor on all other predictors. Interpretation thresholds are: VIF<2.5 (excellent independence), 2.5-5 (acceptable), 5-10 (concerning, requiring intervention), >10 (severe, mandating exclusion). Conservative threshold of VIF≥5 is applied for exclusion decisions. Pairwise correlation coefficients are calculated for all continuous variable pairs, with \|r\|>0.7 flagged as potentially collinear. Sequential exclusion criteria are applied: (1) variables with VIF≥5 are prioritized for exclusion; (2) among correlated pairs (\|r\|>0.7), the variable with higher VIF is excluded preferentially; (3) when VIF values are comparable, clinically essential variables are retained; (4) variables with VIF>10 are excluded regardless of clinical importance. Post-exclusion VIF is recalculated to confirm all retained variables achieve VIF<5 (target: <2.5).* | | | | | | | | |
| *MII oocytes are excluded due to collinearity with both oocytes retrieved (r=0.937) and trigger E2 (r=0.722). 'Excluded' indicates removal during collinearity screening; 'Passed' indicates retention for further evaluation.* | | | | | | | | |
| *Abbreviations: AFC, antral follicle count; AMH, anti-Müllerian hormone; BMI, body mass index; E2, estradiol; FSH, follicle-stimulating hormone; Gn, gonadotropin; ICSI, intracytoplasmic sperm injection; IU, international unit; LH, luteinizing hormone; MII, metaphase II; OPU, oocyte pickup; r, Pearson correlation coefficient; R², coefficient of determination; VIF, variance inflation factor.* | | | | | | | | |
