# Supplementary material for: Nonlinear effects of post-denudation timing on day 3 embryo outcomes in ICSI and evidence for a translatable optimization window
Source: J Transl Med. 2026 Jul 11;24:894. doi: 10.1186/s12967-026-08586-0 (PMC13366850; doi:10.1186/s12967-026-08586-0)
Supplement: Supplementary file 7 — Supplementary Table 3 [file 12967_2026_8586_MOESM7_ESM.docx]

**Table S3. Univariable and multivariable associations of covariates with day 3 embryo utilization rate across progressive model building stages**

|  | | **Univariate Analysis** | | | **Multivariable Analysis (Model 4)** | | |
| --- | --- | --- | --- | --- | --- | --- | --- |
| **Category** | **Variable** | **F** | **P** | **R²** | **F** | **P** | **Partial η²** |
| **Demographics** | Female age (years) | 4.18 | 0.041 | 0.004 | 1.47 | 0.226 | 0.001 |
|  | Female BMI (kg/m²) | 1.68 | 0.196 | 0.001 | 2.89 | 0.090 | 0.003 |
| **Ovarian Reserve** | AMH (ng/mL) | 7.03 | <0.001 | 0.018 | 1.41 | 0.238 | 0.004 |
|  | Basal FSH (IU/L) | 21.99 | <0.001 | 0.019 | 1.52 | 0.218 | 0.001 |
|  | Basal LH (IU/L) | 4.59 | 0.032 | 0.004 | 1.01 | 0.315 | 0.001 |
|  | Basal E2 (pg/mL) | 4.54 | 0.033 | 0.004 | 4.80 | 0.029 | 0.004 |
| **Treatment Factors** | Stimulation protocol | 2.82 | 0.038 | 0.007 | 0.94 | 0.422 | 0.003 |
|  | Stimulation duration (days) | 2.35 | 0.125 | 0.002 | 2.78 | 0.096 | 0.002 |
|  | Total Gn dose (IU) | 7.95 | 0.005 | 0.007 | 4.52 | 0.034 | 0.004 |
|  | E2 at trigger (pg/mL) | 7.53 | <0.001 | 0.019 | 0.75 | 0.521 | 0.002 |
|  | E2 per MII (pg/mL) | 29.15 | <0.001 | 0.025 | 31.45 | <0.001 | 0.027 |
|  | Female ethnicity | 6.39 | 0.012 | 0.006 | 5.88 | 0.015 | 0.005 |
|  | Parity | 2.38 | 0.050 | 0.008 | 3.42 | 0.009 | 0.012 |
|  | Abortions | 1.52 | 0.169 | 0.008 | 1.42 | 0.203 | 0.008 |
|  | Miscarriages | 1.69 | 0.149 | 0.006 | 1.12 | 0.346 | 0.004 |
| **Excluded Variables** | Infertility diagnosis | 0.61 | 0.721 | 0.003 | — | — | — |
|  | Infertility type | 0.40 | 0.525 | 0.000 | — | — | — |
|  | Infertility duration (years) | 0.16 | 0.685 | 0.000 | — | — | — |
|  | Male ethnicity | 0.13 | 0.718 | 0.000 | — | — | — |
| *Univariable analysis results are presented as F-statistic, P-value, and R² for all candidate covariates. Multivariable analysis (Model 4) results are presented as F-statistic, P-value, and Partial η² for retained variables only. Covariates are grouped by categories: Demographics, Ovarian Reserve, Treatment Factors (representing variables included in Model 4), and Excluded Variables (covariates not meeting selection criteria). En-dash (—) in multivariable columns indicates variables excluded from Model 4.* | | | | | | | |
| *Univariable analysis employs simple linear regression for continuous variables (with restricted cubic splines transformation for non-linear relationships identified by generalized additive models) or analysis of variance for categorical variables. Multivariable analysis (Model 4) uses ordinary least squares regression with Type II analysis of variance. For restricted cubic splines-modeled variables in Model 4, overall effect (testing all spline terms jointly) and nonlinear component (testing departure from linearity) are presented. R² represents the proportion of variance in day 3 embryo utilization rate explained by each variable alone in univariable analysis. Partial η² quantifies the unique contribution of each variable after adjusting for all other covariates in Model 4, calculated as Partial sum of squares / (Partial sum of squares + Error sum of squares).* | | | | | | | |
| *Variable selection rationale: Excluded variables fail to meet either statistical criterion (univariable P≥0.20) or clinical necessity for forced inclusion. Multicollinearity diagnostics and VIF-based exclusions are detailed in Table S3. Complete Model 4 specification: d3_utilization ~ rcs(time_denude_to_icsi, 3) + age + bmi + rcs(amh, 4) + b_fsh + b_lh + b_e2 + protocol + stim_days + gn_total + rcs(trig_e2, 4) + e2_per_mii + female_ethnic + parity + abortion + miscarriage. Model performance: R²=0.104, Adjusted R²=0.076, N=1,152, maximum VIF=1.76.* | | | | | | | |
| *Abbreviations: AFC, antral follicle count; AMH, anti-Müllerian hormone; ANOVA, analysis of variance; BMI, body mass index; df, degrees of freedom; E2, estradiol; F, F-statistic; FSH, follicle-stimulating hormone; GAM, generalized additive model; Gn, gonadotropin; hCG, human chorionic gonadotropin; ICSI, intracytoplasmic sperm injection; LH, luteinizing hormone; MII, metaphase II; n, number; OPU, oocyte pickup; P, P-value; R², coefficient of determination; RCS, restricted cubic splines; SS, sum of squares; VIF, variance inflation factor; η², eta-squared.* | | | | | | | |
