# Supplementary material for: Nonlinear effects of post-denudation timing on day 3 embryo outcomes in ICSI and evidence for a translatable optimization window
Source: J Transl Med. 2026 Jul 11;24:894. doi: 10.1186/s12967-026-08586-0 (PMC13366850; doi:10.1186/s12967-026-08586-0)
Supplement: Supplementary file 8 — Supplementary Table 4 [file 12967_2026_8586_MOESM8_ESM.docx]

**Table S4. Progressive model building for day 3 embryo utilization rate across four hierarchical regression models**

|  |  |  | **Overall Performance** | | | | | | | | **Primary Exposure: Overall Time Effect** | | | | | **Primary Exposure: Nonlinear Component** | | | |
| --- | --- | --- | --- | --- | --- | --- | --- | --- | --- | --- | --- | --- | --- | --- | --- | --- | --- | --- | --- |
| **Model** | **Description** | **df** | **R²** | **Adj R²** | **ΔR²** | **AIC** | **BIC** | **Residual SE** | **F** | **P** | **SS** | **MS** | **F** | **P** | **Partial η²** | **SS** | **MS** | **F** | **P** |
| **Model 1** | Time variable only | 2 | 0.008 | 0.006 | — | 163.09 | 183.29 | 0.509 | 4.39 | 0.0126 | 0.590 | 0.295 | 4.39 | 0.0126 | 0.008 | 0.518 | 0.518 | 7.71 | 0.0056 |
| **Model 2** | + Demographics | 4 | 0.012 | 0.008 | 0.004 | 162.07 | 192.36 | 0.509 | 3.46 | 0.0081 | 0.522 | 0.261 | 3.90 | 0.0206 | 0.007 | 0.482 | 0.482 | 7.20 | 0.0074 |
| **Model 3** | + Ovarian reserve | 10 | 0.038 | 0.029 | 0.026 | 143.50 | 204.09 | 0.506 | 4.48 | <0.001 | 0.458 | 0.229 | 3.49 | 0.0308 | 0.006 | 0.453 | 0.453 | 6.92 | 0.0087 |
| **Model 4** | + Treatment variables | 34 | 0.104 | 0.076 | 0.066 | 109.79 | 291.57 | 0.500 | 3.80 | <0.001 | 0.417 | 0.209 | 3.35 | 0.0356 | 0.006 | 0.417 | 0.417 | 6.69 | 0.0098 |
| *Model performance metrics are presented as degrees of freedom (df), R², adjusted R², incremental R² change (ΔR²), information criteria (AIC, BIC), residual standard error, and F-statistics with P-values. Effect sizes for the primary exposure are presented as sum of squares (SS), mean squares (MS), F-statistics, P-values, and partial η².* | | | | | | | | | | | | | | | | | | | |
| *Four hierarchical models are constructed using ordinary least squares regression with Type II ANOVA. Model 1 includes denudation-to-ICSI interval only (modeled with restricted cubic splines, 3 knots at 10th, 50th, 90th percentiles, df=2). Model 2 adds patient demographics (age, BMI). Model 3 adds ovarian reserve markers (AMH with RCS 4 knots, basal FSH, LH, E2). Model 4 adds treatment factors (stimulation protocol, gonadotropin dose, stimulation duration, trigger E2 with RCS 4 knots, ethnicity, parity, abortion history, miscarriage history). Sample size is N=1,152 ICSI cycles across all models. ΔR² represents incremental variance explained by each variable group relative to the previous model. Partial η² represents the proportion of outcome variance uniquely explained by the time variable after controlling for all other predictors in the model.* | | | | | | | | | | | | | | | | | | | |
| *Abbreviations: Adj R², adjusted coefficient of determination; AIC, Akaike Information Criterion; AMH, anti-Müllerian hormone; ANOVA, analysis of variance; BIC, Bayesian Information Criterion; BMI, body mass index; df, degrees of freedom; E2, estradiol; F, F-statistic from ANOVA; FSH, follicle-stimulating hormone; ICSI, intracytoplasmic sperm injection; LH, luteinizing hormone; MS, mean square; P, P-value; R², coefficient of determination; RCS, restricted cubic splines; Residual SE, residual standard error; SS, sum of squares.* | | | | | | | | | | | | | | | | | | | |
