# Supplementary material for: Nonlinear effects of post-denudation timing on day 3 embryo outcomes in ICSI and evidence for a translatable optimization window
Source: J Transl Med. 2026 Jul 11;24:894. doi: 10.1186/s12967-026-08586-0 (PMC13366850; doi:10.1186/s12967-026-08586-0)
Supplement: Supplementary file 9 — Supplementary Table 5 [file 12967_2026_8586_MOESM9_ESM.docx]

**Table S5. Stepwise backward elimination process using Bayesian information criterion for model optimization**

|  |  |  | **Model 4 Statistics** | | **BIC Metrics** | |  | **Cumulative Progress** | |  |
| --- | --- | --- | --- | --- | --- | --- | --- | --- | --- | --- |
| **Phase** | **Step** | **Variable** | **P-value** | **F** | **Post-removal BIC** | **ΔBIC** | **N Vars Remaining** | **Total ΔBIC** | **Improvement (%)** | **Removal Rationale** |
| **Initial State** | **0 (Start)** | (Initial Model) | <0.001 | 3.80 | 291.6 | — | 16 | — | — | Baseline |
| **Major Elimination (Steps 1-5)** | **1** | Abortions | 0.203 | 1.42 | 258.0 | -33.5 | 15 | -33.5 | 11.5% | \|ΔBIC\|≥10; NS |
|  | **2** | Miscarriages | 0.346 | 1.12 | 234.5 | -23.5 | 14 | -57.0 | 19.6% | \|ΔBIC\|≥10; NS |
|  | **3** | E2 at trigger | 0.521 | 0.75 | 215.7 | -18.8 | 13 | -75.8 | 26.0% | \|ΔBIC\|≥10; NS |
|  | **4** | Stimulation protocol | 0.422 | 0.94 | 197.4 | -18.3 | 12 | -94.1 | 32.3% | \|ΔBIC\|≥10; NS |
|  | **5** | Parity | 0.009 | 3.42 | 182.1 | -15.3 | 11 | -109.4 | 37.5% | \|ΔBIC\|≥10 |
| **Fine-tuning (Steps 6-12)** | **6** | AMH | 0.238 | 1.41 | 174.2 | -7.9 | 10 | -117.3 | 40.2% | 5≤\|ΔBIC\|<10; NS |
|  | **7** | Basal LH | 0.315 | 1.01 | 167.2 | -7.0 | 9 | -124.4 | 42.7% | 5≤\|ΔBIC\|<10; NS |
|  | **8** | Stimulation duration | 0.096 | 2.78 | 160.5 | -6.7 | 8 | -131.1 | 45.0% | 5≤\|ΔBIC\|<10; NS |
|  | **9** | Basal E2 | 0.029 | 4.80 | 154.3 | -6.2 | 7 | -137.3 | 47.1% | 5≤\|ΔBIC\|<10 |
|  | **10** | Female BMI | 0.090 | 2.89 | 149.6 | -4.7 | 6 | -142.0 | 48.7% | \|ΔBIC\|<5; NS |
|  | **11** | Female age | 0.226 | 1.47 | 145.2 | -4.3 | 5 | -146.3 | 50.2% | \|ΔBIC\|<5; NS |
|  | **12** | Female ethnicity | 0.015 | 5.88 | 144.6 | -0.6 | 4 | -146.9 | 50.4% | \|ΔBIC\|<5 |
| **Final Retained Variables** |  | Denudation to ICSI interval | 0.036 | 3.35 | 144.6 | — | 4 | — | — | Retained |
|  |  | Basal FSH | 0.218 | 1.52 | 144.6 | — | 4 | — | — | Retained |
|  |  | E2 per MII | <0.001 | 31.45 | 144.6 | — | 4 | — | — | Retained |
|  |  | Total Gn dose | 0.034 | 4.52 | 144.6 | — | 4 | — | — | Retained |
| *Each step in the backward elimination process is presented with the following information: variable removed, P-value and F-statistic from initial Model 4, post-removal BIC value, BIC change (ΔBIC), number of variables remaining, cumulative BIC reduction, percentage improvement, and removal rationale. Variables are grouped into phases based on elimination sequence: Initial State, Major Elimination (Steps 1-5), Fine-tuning (Steps 6-12), and Final Retained Variables.* | | | | | | | | | | |
| *Stepwise backward elimination is performed using Bayesian information criterion (BIC) to optimize model parsimony. Variables are removed sequentially based on the largest BIC reduction at each step, with BIC penalty k=log(n) applied for model complexity. Removal rationale categories are defined as: \|ΔBIC\|≥10 indicates strong improvement; 5≤\|ΔBIC\|<10 indicates moderate improvement; \|ΔBIC\|<5 indicates marginal improvement. The suffix "NS" (non-significant, P≥0.05) is added when the variable was not statistically significant in initial Model 4, indicating removal is particularly justified. Absence of "NS" indicates the variable was significant (P<0.05) but removed based on BIC optimization. The final optimized model retains 4 variables: denudation to ICSI interval, basal FSH, E2 per MII, and total Gn dose.* | | | | | | | | | | |
| *All delta (Δ) values are computed from original full-precision numerical values and then rounded to one decimal place for display.* | | | | | | | | | | |
| *Abbreviations: AMH, anti-Müllerian hormone; ART, assisted reproductive technology; BIC, Bayesian information criterion; E2, estradiol; F, F-statistic; FSH, follicle-stimulating hormone; Gn, gonadotropin; ICSI, intracytoplasmic sperm injection; LH, luteinizing hormone; MII, metaphase II; NS, non-significant; ΔBIC, change in BIC.* | | | | | | | | | | |
