# Supplementary material for: Nonlinear effects of post-denudation timing on day 3 embryo outcomes in ICSI and evidence for a translatable optimization window
Source: J Transl Med. 2026 Jul 11;24:894. doi: 10.1186/s12967-026-08586-0 (PMC13366850; doi:10.1186/s12967-026-08586-0)
Supplement: Supplementary file 10 — Supplementary Table 6 [file 12967_2026_8586_MOESM10_ESM.docx]

**Table S6. Dual-criteria variable selection from Model 4 to final optimized model using BIC and LASSO methods**

| **Category** | **Variable** | **BIC** | **LASSO** | **Coefficient Status** | **Final Status** |
| --- | --- | --- | --- | --- | --- |
| **Demographics** | Female age | Excluded | Excluded | Penalized | Excluded |
|  | Female BMI | Excluded | Excluded | Penalized | Excluded |
|  | Female ethnicity | Excluded | Excluded | Penalized | Excluded |
| **Reproductive History** | Parity | Excluded | Excluded | Penalized | Excluded |
|  | Abortions | Excluded | Excluded | Penalized | Excluded |
|  | Miscarriages | Excluded | Excluded | Penalized | Excluded |
| **Ovarian Reserve** | AMH | Excluded | Excluded | Penalized | Excluded |
|  | Basal FSH | Selected | Selected | 0.0011 | Included |
|  | Basal LH | Excluded | Excluded | Penalized | Excluded |
|  | Basal E2 | Excluded | Excluded | Penalized | Excluded |
| **Treatment Factors** | Stimulation protocol | Excluded | Excluded | Penalized | Excluded |
|  | Stimulation duration | Excluded | Excluded | Penalized | Excluded |
|  | Total Gn dose | Selected | Excluded | Penalized | Excluded |
|  | E2 at trigger | Excluded | Excluded | Penalized | Excluded |
|  | E2 per MII oocyte | Selected | Selected | Non-zero* | Included |
| *The table presents dual-criteria variable selection results from the Model 4 covariate pool to the final optimized model. For each variable, four columns are displayed: (1) BIC—retention status under Bayesian Information Criterion stepwise backward elimination (Selected/Excluded); (2) LASSO—retention status under Elastic Net regularization (Selected/Excluded); (3) Coefficient Status—penalized coefficient values from LASSO (numeric values for non-zero coefficients, 'Penalized' for coefficients shrunk to zero); (4) Final Status—inclusion decision based on inter-method consensus (Included/Excluded). A total of 15 covariates across four categories are evaluated: Demographics (n=3), Reproductive History (n=3), Ovarian Reserve (n=4), and Treatment Factors (n=5). The primary exposure variable (denudation-to-ICSI time interval) is mandatorily retained throughout all selection steps and is not displayed in this table.* | | | | | |
| *Two independent variable selection methods are applied to 15 covariates from the Model 4 pool: (A) BIC-based stepwise backward elimination (penalty factor k=log(n)=7.05, conservative approach favoring model simplicity) selects 3 covariates; (B) Elastic Net regularization (α=0.5 balancing L1 LASSO and L2 Ridge penalties, optimal λ=0.061804 determined by 10-fold cross-validation with 1-SE rule) selects 2 covariates. Inter-method consistency is 66.7% (2/3 covariates selected by both methods). The final model adopts an intersection approach, retaining only covariates selected by both methods, resulting in 2 covariates plus the primary exposure (3 total predictors). Decision terminology: Selected—retained by the respective method; Excluded—removed by the method. Final Status: Included—selected by both BIC and LASSO (strongest evidence); Excluded—selected by fewer than 2 methods.* | | | | | |
| **Non-zero coefficient with \|coef\|<0.0001 in LASSO design matrix (may include RCS basis functions or factor dummy variables).* | | | | | |
| *Abbreviations: AMH, anti-Müllerian hormone; BIC, Bayesian Information Criterion; BMI, body mass index; CV, cross-validation; E2, estradiol; FSH, follicle-stimulating hormone; Gn, gonadotropin; ICSI, intracytoplasmic sperm injection; LASSO, least absolute shrinkage and selection operator; LH, luteinizing hormone; MII, metaphase II; RCS, restricted cubic splines.* | | | | | |
