# Supplementary material for: Nonlinear effects of post-denudation timing on day 3 embryo outcomes in ICSI and evidence for a translatable optimization window
Source: J Transl Med. 2026 Jul 11;24:894. doi: 10.1186/s12967-026-08586-0 (PMC13366850; doi:10.1186/s12967-026-08586-0)
Supplement: Supplementary file 11 — Supplementary Table 7 [file 12967_2026_8586_MOESM11_ESM.docx]

**Table S7. Interaction effect tests between DTI and patient/treatment characteristics for day 3 embryo utilization rate**

| **Tier** | **Interaction** | **Base R²** | **Int R²** | **ΔR²** | **LRT χ²** | **df** | **P-value** | **FDR Q** | **ΔAIC** | **ΔBIC** | **Result** |
| --- | --- | --- | --- | --- | --- | --- | --- | --- | --- | --- | --- |
| **Tier 1** | Time × Basal FSH | 0.0504 | 0.0507 | +0.0003 | 0.02 | 1 | 0.5559 | 0.5559 | +1.7 | +6.7 | NS |
|  | Time × E2 per MII Oocyte | 0.0504 | 0.0512 | +0.0008 | 0.06 | 1 | 0.3185 | 0.5559 | +1.0 | +6.0 | NS |
| **Tier 2** | Time × Age | 0.0518 | 0.0520 | +0.0002 | 0.02 | 1 | 0.6058 | 0.9902 | +2.1 | +12.2 | NS |
|  | Time × Age Group | 0.0514 | 0.0514 | +0.0000 | 0.00 | 2 | 0.9902 | 0.9902 | +6.8 | +27.0 | NS |
|  | Time × AMH | 0.0523 | 0.0523 | +0.0000 | 0.00 | 1 | 0.8915 | 0.9902 | +1.7 | +11.8 | NS |
|  | Time × AMH Group | 0.0562 | 0.0574 | +0.0011 | 0.09 | 2 | 0.5050 | 0.9902 | -0.5 | +19.7 | NS |
|  | Time × Ovarian Response | 0.0885 | 0.0913 | +0.0028 | 0.21 | 3 | 0.3271 | 0.9902 | -38.6 | -8.3 | NS |
| **Tier 3** | Time × HCG to OPU Interval | 0.0523 | 0.0533 | +0.0010 | 0.08 | 1 | 0.2768 | 0.3762 | +0.6 | +10.7 | NS |
|  | Time × OPU to Denude Interval | 0.0518 | 0.0525 | +0.0006 | 0.05 | 1 | 0.3762 | 0.3762 | +1.5 | +11.6 | NS |
| **Tier 4** | Time × BMI | 0.0533 | 0.0534 | +0.0001 | 0.01 | 1 | 0.7710 | 0.9285 | +0.4 | +10.5 | NS |
|  | Time × BMI Group | 0.0583 | 0.0590 | +0.0007 | 0.06 | 2 | 0.6348 | 0.9285 | -2.5 | +17.7 | NS |
|  | Time × AFC | 0.0538 | 0.0538 | +0.0000 | 0.00 | 1 | 0.9285 | 0.9285 | -0.1 | +10.0 | NS |
|  | Time × AFC Group | 0.0567 | 0.0579 | +0.0012 | 0.09 | 2 | 0.4954 | 0.9285 | -1.1 | +19.1 | NS |
|  | Time × Stimulation Protocol | 0.0578 | 0.0618 | +0.0040 | 0.31 | 3 | 0.1816 | 0.9081 | -1.9 | +28.4 | NS |
| *Data are presented as R² values, change in R² (ΔR²), likelihood ratio test (LRT) statistics with degrees of freedom (df), P-values, false discovery rate-adjusted Q-values (FDR Q), and information criteria changes (ΔAIC, ΔBIC). Interaction terms are tested across four hierarchical tiers based on clinical relevance and model priority.* | | | | | | | | | | | |
| *Interaction effects are tested using hierarchical linear regression models comparing base model (d3_utilization ~ rcs(time_denude_to_icsi, 3) + b_fsh + e2_per_mii) against interaction-augmented models. LRT is used to assess statistical significance with tier-specific thresholds: Tier 1 (model-internal variables) and Tier 4 (secondary exploratory) use P<0.10 and FDR Q<0.15; Tier 2 (clinical key variables including age, AMH, and ovarian response) and Tier 3 (time window variables) use stringent P<0.05 and FDR Q<0.10. Information criteria (AIC, BIC) are calculated relative to base model, with ΔAIC<-2 or ΔBIC<-2 indicating preference for interaction model. FDR correction is applied using Benjamini-Hochberg method to control for multiple testing.* | | | | | | | | | | | |
| *ΔR² represents change in explained variance when adding interaction term, with ΔR²≥0.01 suggesting clinically relevant effect size regardless of statistical significance. ΔAIC and ΔBIC are calculated as (interaction model criterion) - (base model criterion); negative values favor interaction model. NS indicates not significant (FDR Q>threshold); SIG indicates significant (FDR Q≤threshold). Primary finding: no significant interactions are detected (0/14, all FDR Q>0.10), indicating stable time effect across patient subgroups and supporting unified clinical application without stratification.* | | | | | | | | | | | |
| *Abbreviations: AFC, antral follicle count; AIC, Akaike information criterion; AMH, anti-Müllerian hormone; BIC, Bayesian information criterion; BMI, body mass index; df, degrees of freedom; E2, estradiol; FDR, false discovery rate; FSH, follicle-stimulating hormone; HCG, human chorionic gonadotropin; ICSI, intracytoplasmic sperm injection; LRT, likelihood ratio test; MII, metaphase II; NS, not significant; OPU, oocyte pickup; rcs, restricted cubic spline; SIG, significant.* | | | | | | | | | | | |
