# Supplementary material for: Nonlinear effects of post-denudation timing on day 3 embryo outcomes in ICSI and evidence for a translatable optimization window
Source: J Transl Med. 2026 Jul 11;24:894. doi: 10.1186/s12967-026-08586-0 (PMC13366850; doi:10.1186/s12967-026-08586-0)
Supplement: Supplementary file 12 — Supplementary Table 8 [file 12967_2026_8586_MOESM12_ESM.docx]

**Table S8. Subgroup-specific effects of DTI on day 3 embryo utilization rate stratified by patient and treatment characteristics**

| **Stratification** | **Subgroup** | **N** | **β Coefficient** | **95% CI** | **F Value** | **P Value** | **R²** |
| --- | --- | --- | --- | --- | --- | --- | --- |
| **Age Group** | <35 years | 748 | 0.049 | [0.015, 0.084] | F(2, 744) = 3.88 | 0.021 | 8.51% |
|  | 35-37 years | 185 | -0.010 | [-0.085, 0.065] | F(2, 181) = 0.07 | 0.934 | 3.22% |
|  | ≥38 years | 219 | 0.064 | [-0.030, 0.158] | F(2, 215) = 1.67 | 0.191 | 2.60% |
| **AMH Group** | <1.2 ng/mL | 202 | 0.017 | [-0.098, 0.132] | F(2, 198) = 1.23 | 0.295 | 2.20% |
|  | 1.2-3.5 ng/mL | 473 | 0.014 | [-0.033, 0.061] | F(2, 469) = 0.74 | 0.479 | 7.38% |
|  | >3.5 ng/mL | 477 | 0.053 | [0.014, 0.091] | F(2, 473) = 3.61 | 0.028 | 6.58% |
| **Ovarian Response** | Poor (≤3 oocytes) | 166 | 0.036 | [-0.110, 0.182] | F(2, 162) = 0.42 | 0.658 | 2.56% |
|  | Suboptimal (4-9) | 342 | -0.025 | [-0.078, 0.029] | F(2, 338) = 1.91 | 0.149 | 7.10% |
|  | Normal (10-15) | 336 | 0.045 | [-0.002, 0.092] | F(2, 332) = 1.92 | 0.148 | 8.89% |
|  | High (>15) | 308 | 0.042 | [-0.005, 0.089] | F(2, 304) = 2.06 | 0.129 | 11.01% |
| **Stimulation Protocol** | GnRH Agonist | 428 | 0.067 | [0.017, 0.117] | F(2, 424) = 4.48 | 0.012 | 4.86% |
|  | GnRH Antagonist | 340 | 0.071 | [0.012, 0.130] | F(2, 336) = 2.76 | 0.064 | 5.93% |
|  | Progestin-Primed | 357 | 0.014 | [-0.047, 0.074] | F(2, 353) = 0.33 | 0.721 | 7.51% |
| **BMI Group** | <18.5 kg/m² | 90 | 0.058 | [-0.070, 0.185] | F(2, 86) = 0.80 | 0.453 | 4.52% |
|  | 18.5-24.9 kg/m² | 817 | 0.028 | [-0.008, 0.064] | F(2, 813) = 1.15 | 0.316 | 5.07% |
|  | ≥25 kg/m² | 245 | 0.086 | [0.024, 0.148] | F(2, 241) = 4.31 | 0.014 | 8.16% |
| **AFC Group** | <5 | 133 | 0.034 | [-0.105, 0.173] | F(2, 129) = 0.79 | 0.457 | 1.56% |
|  | 5-15 | 566 | 0.019 | [-0.025, 0.063] | F(2, 562) = 0.44 | 0.644 | 5.56% |
|  | >15 | 453 | 0.055 | [0.015, 0.095] | F(2, 449) = 4.03 | 0.018 | 7.31% |
| *Data are presented as β coefficients with 95% confidence intervals (CI), F statistics in the format F(df1, df2), P values, and R² values for each subgroup. The β coefficient represents the effect of denudation-to-ICSI interval on day 3 embryo utilization rate. Six stratification variables are analyzed: Age Group (ESHRE criteria: <35, 35-37, ≥38 years), AMH Group (POSEIDON criteria: <1.2, 1.2-3.5, >3.5 ng/mL), Ovarian Response (Bologna criteria based on oocyte yield: Poor ≤3, Suboptimal 4-9, Normal 10-15, High >15), Stimulation Protocol (GnRH Agonist, Antagonist, Progestin-Primed), BMI Group (WHO criteria: <18.5, 18.5-24.9, ≥25 kg/m²), and AFC Group (POSEIDON criteria: <5, 5-15, >15).* | | | | | | | |
| *All subgroup models use the same specification as the final optimized model: d3_utilization ~ rcs(time_denude_to_icsi, 3) + b_fsh + e2_per_mii, where time-to-ICSI is modeled using restricted cubic splines with 3 knots to capture potential nonlinear effects. The F statistic evaluates the joint significance of the time-to-ICSI effect, with df1=2 representing the numerator degrees of freedom for the two RCS components (linear and nonlinear terms), and df2 representing the denominator degrees of freedom calculated as subgroup sample size minus 4 (N-4, accounting for the intercept and three predictors). R² indicates the proportion of variance explained by the full model.* | | | | | | | |
| *The Mild protocol group (n=27) is excluded from analysis due to insufficient sample size (<30), resulting in 19 subgroups analyzed. Five of 19 subgroups (26.3%) show statistically significant effects: Age <35 years, AMH >3.5 ng/mL, GnRH Agonist protocol, BMI ≥25 kg/m², and AFC >15. Notably, 17 subgroups (89.5%) exhibit consistent positive effect direction. The absence of statistical significance in 14 subgroups reflects insufficient statistical power rather than true null effects. Formal interaction analysis (Table S9) confirms no significant interactions between time-to-ICSI and any stratification variable after FDR correction for multiple testing (all FDR Q>0.10), supporting effect stability across patient subgroups and the clinical applicability of unified time management recommendations.* | | | | | | | |
| *Abbreviations: AFC, antral follicle count; AMH, anti-Müllerian hormone; BMI, body mass index; CI, confidence interval; df, degrees of freedom; ESHRE, European Society of Human Reproduction and Embryology; FDR, false discovery rate; GnRH, gonadotropin-releasing hormone; ICSI, intracytoplasmic sperm injection; POSEIDON, Patient-Oriented Strategies Encompassing IndividualizeD Oocyte Number; RCS, restricted cubic spline; WHO, World Health Organization.* | | | | | | | |
