# Supplementary material for: Nonlinear effects of post-denudation timing on day 3 embryo outcomes in ICSI and evidence for a translatable optimization window
Source: J Transl Med. 2026 Jul 11;24:894. doi: 10.1186/s12967-026-08586-0 (PMC13366850; doi:10.1186/s12967-026-08586-0)
Supplement: Supplementary file 13 — Supplementary Table 9 [file 12967_2026_8586_MOESM13_ESM.docx]

**Table S9. Heterogeneity statistics for DTI effect on day 3 embryo utilization rate across patient and treatment subgroups**

| **Stratification Variable** | **N Subgroups** | **Cochran's Q** | **df** | **P Value** | **I² (%)** | **τ²** | **Heterogeneity Level** |
| --- | --- | --- | --- | --- | --- | --- | --- |
| Age Group | 3 | 2.24 | 2 | 0.326 | 10.7% | 0.0001 | Low |
| AMH Group | 3 | 1.67 | 2 | 0.433 | 0% | <0.0001 | Low |
| Ovarian Response | 4 | 4.56 | 3 | 0.207 | 34.2% | 0.0005 | Moderate |
| Stimulation Protocol | 3 | 2.27 | 2 | 0.322 | 11.7% | 0.0001 | Low |
| BMI Group | 3 | 2.56 | 2 | 0.278 | 21.8% | 0.0003 | Low |
| AFC Group | 3 | 1.45 | 2 | 0.485 | 0% | <0.0001 | Low |
| *Data are presented as N Subgroups indicates the number of strata within each stratification variable; Cochran's Q and degrees of freedom (df) are presented as integers; P values are presented to three decimal places; I² (percentage of variation attributable to heterogeneity) is presented as a percentage; τ² (between-subgroup variance) is presented to four decimal places; Heterogeneity Level is categorized based on I² thresholds.* | | | | | | | |
| *Heterogeneity across subgroups is assessed using Cochran's Q test, I² statistic, and τ² estimate. Cochran's Q follows a chi-squared distribution with degrees of freedom (df) = number of subgroups - 1, testing whether subgroup effects are homogeneous. I² quantifies the percentage of total variation across subgroups due to true heterogeneity rather than sampling error, calculated as I² = max(0, [(Q - df)/Q] × 100%). τ² represents the between-subgroup variance estimated using the DerSimonian-Laird method. Heterogeneity levels are classified as: Low (I²<25%), Moderate (I²=25-50%), High (I²=50-75%), or Very High (I²>75%).* | | | | | | | |
| *Five of six stratifications (83.3%) show low heterogeneity (I²<25%), indicating stable time-to-ICSI effects across patient subgroups. Only Ovarian Response exhibits moderate heterogeneity (I²=34.2%, Q=4.56, P=0.207), though the Q test remains non-significant, suggesting the observed variation is likely due to random sampling error rather than true effect modification. All six stratifications have non-significant Q tests (all P>0.05), and the absence of significant interactions in formal testing (Table S7, all FDR Q>0.10) further supports the consistency of time effects across subgroups. These findings demonstrate the robustness and generalizability of the time-to-ICSI effect, supporting unified clinical recommendations without stratification.* | | | | | | | |
| *Abbreviations: AFC, antral follicle count; AMH, anti-Müllerian hormone; BMI, body mass index; df, degrees of freedom; FDR, false discovery rate; ICSI, intracytoplasmic sperm injection.* | | | | | | | |
