# Supplementary material for: Nonlinear effects of post-denudation timing on day 3 embryo outcomes in ICSI and evidence for a translatable optimization window
Source: J Transl Med. 2026 Jul 11;24:894. doi: 10.1186/s12967-026-08586-0 (PMC13366850; doi:10.1186/s12967-026-08586-0)
Supplement: Supplementary file 14 — Supplementary Table 10 [file 12967_2026_8586_MOESM14_ESM.docx]

**Table S10. Comprehensive robustness analysis summary with component-wise scores and overall rating**

| **Dimension** | **Criterion** | **Excellence Threshold** | **Observed Value** | **Score** | **Weight** | **Weighted Score** | **Rating** |
| --- | --- | --- | --- | --- | --- | --- | --- |
| **Statistical Inference** | Weighted Bootstrap Coefficient CV | CV < 0.10 | 0.1403 | 6/10 | 20% | 1.20 | Fair |
|  | Robust Standard Error Consistency | SE change < 10% | 10.02% | 7/10 | 10% | 0.70 | Good |
|  | Confidence Interval Consistency | All 3 CIs exclude zero | 100.00% | 10/10 | 10% | 1.00 | Excellent |
|  | Subtotal (3 criteria) | ― | ― | 7.3/10 | 40% | 2.90 | Good |
| **Data Robustness** | Influential Point Deletion Stability | Significant after removing 8% influential points | 100.00% | 10/10 | 15% | 1.50 | Excellent |
|  | Extreme Value Treatment Stability | Significant after 5% Winsorization | 100.00% | 10/10 | 10% | 1.00 | Excellent |
|  | Random Subsampling Significance Retention | Retention rate > 95% | 92.00% | 8/10 | 15% | 1.20 | Very Good |
|  | Subtotal (3 criteria) | ― | ― | 9.2/10 | 40% | 3.70 | Excellent |
| **Prediction Stability** | LOOCV Absolute R² Loss | Absolute loss < 0.01 | 0.0097 | 8/10 | 10% | 0.80 | Very Good |
|  | K-Fold CV Absolute R² Loss | Absolute loss < 0.01 | 0.0082 | 8/10 | 10% | 0.80 | Very Good |
|  | Subtotal (2 criteria) | ― | ― | 8.0/10 | 20% | 1.60 | Very Good |
| **Overall** | Total (8 criteria) | ― | ― | 8.2/10 | 100% | 8.20 | High Robustness |
| *Robustness assessment is presented across eight criteria grouped into three dimensions: Statistical Inference (3 criteria, 40% weight), Data Robustness (3 criteria, 40% weight), and Prediction Stability (2 criteria, 20% weight). Each criterion is evaluated using excellence thresholds, observed values, individual scores (0-10 scale), assigned weights, weighted scores, and qualitative ratings. Overall robustness is calculated as the weighted sum of component scores.* | | | | | | | |
| *Statistical inference robustness is assessed through bootstrap resampling (B=2,000 iterations) to evaluate coefficient stability via weighted coefficient of variation (CV), heteroskedasticity-consistent standard errors (HC3 estimator) to verify inference validity under potential heteroskedasticity, and consistency across three confidence interval methods (percentile, BCa, and normal approximation). Data robustness is examined by testing model stability after removing influential observations identified through multiple diagnostics (Cook's distance, DFBETAS, DFFITS, hat values, and studentized residuals), evaluating sensitivity to extreme values through 5% two-sided Winsorization, and assessing coefficient significance retention across 100 random 80% subsamples. Prediction stability is measured using leave-one-out cross-validation (LOOCV) and 10-fold cross-validation (repeated 5 times) to evaluate absolute R² loss and generalizability to new data.* | | | | | | | |
| *The overall robustness score of 8.20/10.00 (82.0%) indicates high robustness. The main exposure effect (denudation-to-ICSI interval) remains statistically significant across all robustness tests, with 100% confidence interval consistency, 100% stability after influential point deletion, 100% stability after extreme value treatment, and 92% significance retention in random subsampling.* | | | | | | | |
| *Abbreviations: BCa, bias-corrected and accelerated; CI, confidence interval; CV, coefficient of variation; DFBETAS, standardized difference in coefficients; DFFITS, standardized difference in fitted values; HC3, heteroskedasticity-consistent type 3; LOOCV, leave-one-out cross-validation; SE, standard error.* | | | | | | | |
